# Molecular simulation of lignin-related aromatic compound permeation through gram-negative bacterial outer membranes

**DOI:** 10.1016/j.jbc.2022.102627

**Published:** 2022-10-21

**Authors:** Josh V. Vermaas, Michael F. Crowley, Gregg T. Beckham

**Affiliations:** 1 Biosciences Center, National Renewable Energy Laboratory, Golden, Colorado, USA; 2 National Center for Computational Sciences, Oak Ridge National Laboratory, Oak Ridge, Tennessee, USA; 3 MSU-DOE Plant Research Laboratory, Michigan State University, East Lansing, Michigan, USA; 4 Department of Biochemistry and Molecular Biology, Michigan State University, East Lansing, Michigan, USA; 5 Renewable Resources and Enabling Sciences Center, National Renewable Energy, Laboratory, Golden, Colorado, USA

**Keywords:** lignin degradation, molecular dynamics, membrane transport, lipopolysaccharide, passive permeation, ABF, adaptive biasing force, IM, inner membrane, LPS, lipopolysaccharide, LRC, lignin related compound, OM, outer membrane, REUS, replica exchange umbrella sampling

## Abstract

Lignin, an abundant aromatic heteropolymer in secondary plant cell walls, is the single largest source of renewable aromatics in the biosphere. Leveraging this resource for renewable bioproducts through targeted microbial action depends on lignin fragment uptake by microbial hosts and subsequent enzymatic action to obtain the desired product. Recent computational work has emphasized that bacterial inner membranes are permeable to many aromatic compounds expected from lignin depolymerization processes. In this study, we expand on these findings through simulations for 42 lignin-related compounds across a gram-negative bacterial outer membrane model. Unbiased simulation trajectories indicate that spontaneous crossing for the full outer membrane is relatively rare at molecular simulation timescales, primarily due to preferential membrane partitioning and slow diffusion within the lipopolysaccharide layer within the outer membrane. Membrane partitioning and permeability coefficients were determined through replica exchange umbrella sampling simulations to overcome sampling limitations. We find that the glycosylated lipopolysaccharides found in the outer membrane increase the permeation barrier to many lignin-related compounds, particularly the most hydrophobic compounds. However, the effect is relatively modest; at industrially relevant concentrations, uncharged lignin-related compounds will readily diffuse across the outer membrane without the need for specific porins. Together, our results provide insight into the permeability of the bacterial outer membrane for assessing lignin fragment uptake and the future production of renewable bioproducts.

Lignin, an aromatic heteropolymer found in terrestrial plant cell walls, is the largest single source of aromatics in the biosphere (1). The substituted aromatics represent a high energy investment during biosynthesis, with substantial opportunities for valorization (2). Utilizing these abundant lignin resources industrially to supplant fossil-derived products would be significant for sustainable economic development through a circular bioeconomy. In nature, fungal and bacterial enzymes breakdown the lignin polymer into smaller fragments (3, 4).

Once fragmented, biological conversion for lignin and lignin related compounds (LRCs) through funneling into targeted end products has substantial promise to valorize lignin and other waste aromatics (5, 6, 7, 8, 9). Aromatic-catabolic bacteria, such as the soil bacterium *Pseudomonas putida*, have substantial metabolic flexibility to utilize an array of LRC molecules in their environment as their initial feedstock toward building designer products (7, 10, 11, 12). This flexibility could be enhanced further by integrating fungal pathways for lignin catabolism into microbial systems (13).

One underexplored aspect to support this work is how LRCs permeate into bacterial cells. *P. putida* and other gram-negative bacteria have two membranes, an inner membrane (IM) composed of phospholipids and an outer membrane (OM) that features a lipopolysaccharide (LPS) membrane leaflet (14). For these bacteria to catabolize LRCs, the compounds must traverse both of these membranes, either passively or *via* transport protein, and also diffuse through the peptidoglycan cell wall.

Past computational work has demonstrated that uncharged LRCs passively permeate through a bacterial IM at rates commensurate with cellular metabolism (15). Additionally, vesicles that bud from the OM have been observed to catalyze lignin catabolism, suggesting that LRCs permeate the OM through an unresolved mechanism (16). The OM LPS glycosylations within the outer leaflet form a hydrophilic region that could act as a permeation barrier to largely hydrophobic LRCs. If the permeation barrier is significant, transport proteins would be required to facilitate LRC flux across the OM. Alternatively, LRCs may passively permeate across the OM, just as was predicted for the IM (15) and recently validated experimentally for plant-like membranes (17).

Through molecular simulation tools, we test the passive permeation hypothesis across the OM by quantifying the permeability for the OM of *P. putida*. Molecular dynamics (MD) is a useful tool for studying small molecule interactions with lipid bilayers (18) and comes with well-developed theory for determining permeability *in silico* (19). While software for building LPS models has only recently become available (20), molecular simulation has quickly proven to be an effective tool for studying LPS-bearing membranes, providing a detailed view into the molecular interactions within LPS and its environment (21, 22).

Through equilibrium and nonequilibrium MD simulations for selected LRC compounds (Fig. 1), we determine that the OM can be a bigger barrier to permeation than the IM, particularly for the most lipophilic LRCs tested. However, the barrier is not sufficiently large to require protein-specific transport processes. The OM slows permeation by a few orders of magnitude compared to IM permeation, with the resulting permeability coefficients remaining high enough to support robust flux across the OM. The LPS region of the OM was found to hinder permeation the most, as the slow LPS dynamics reduce diffusion in that region significantly. Taken together, the results suggest that specific transport proteins are not required for most LRCs to permeate the OM within a biorefinery context, as potential passive fluxes in these scenarios exceed cellular LRC catabolic capacity.

**Figure 1:**
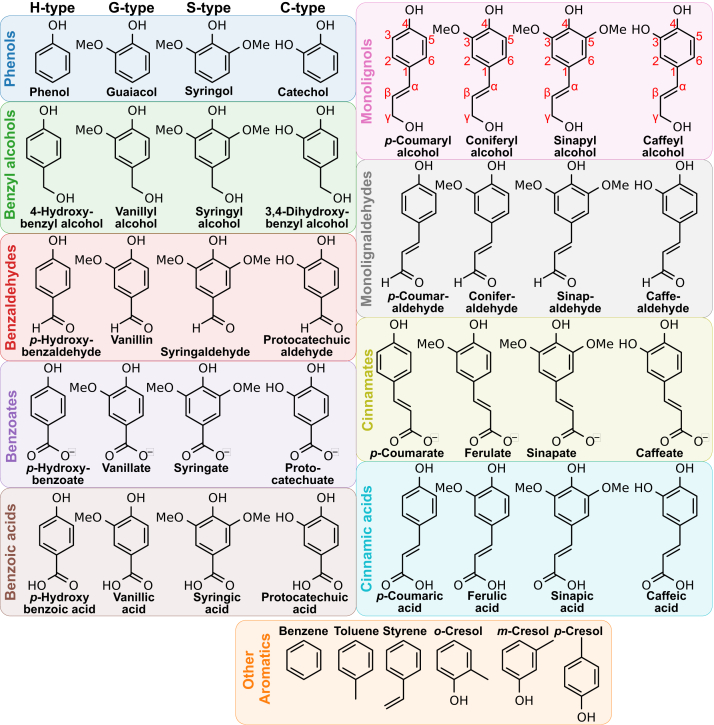
**Lignin-related compounds used in this study.** The aromatic molecules tested represent monomeric lignin compounds with diverse lignin functionalities. Each colored box groups together molecule functionality at position 1 of the aromatic ring. The *box color* is consistent with the color assigned to these functional groups in other Figures. Within the *colored boxes*, the lignin compounds vary based on hydroxylation and methoxylation patterns for H-, G-, S-, and C-type lignin molecules.

## Results

### Equilibrium analysis

**Figure 2:**
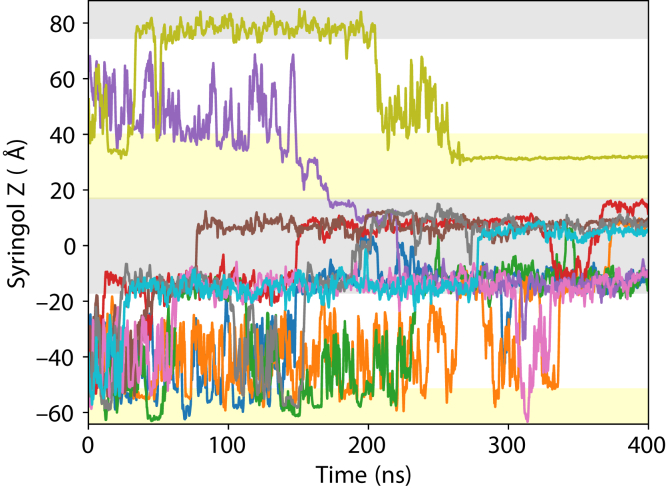
**Traces for the 10 syringol molecules placed into a membrane context.** Each uniquely colored traces mark the pathway for a single syringol molecule, drawn to correct for trajectories that cross the periodic boundary. To assist the reader in tracking the motion across the different compartments, the hydrophobic lipid core region of the membrane has a *gray* background, while the glycosylated region of the membrane has a *yellow* background color. Regions of aqueous solution have no background coloration. The *purple* trace is observed to permeate from the top of the glycolipid leaflet and cross the hydrophobic membrane thickness, briefly even entering solution on the far side.

The unbiased equilibrium trajectories can provide insight into the typical behavior for small molecules near our OM model that is simultaneously glycosylated and lipidated. While individual small molecules can have radically different traces based on the stochastic processes underlying membrane insertion and translocation (Figs. S1–S41), across all 42 compounds under study, we observe only a single instance where a small molecule passively permeates across the OM model generated here within our 400 ns simulation duration. For the syringol system, a single syringol molecule (purple line, Fig. 2) is observed to enter into the glycosylated region marking the OM boundary. Approximately 150 ns into the simulation, this single syringol molecule enters more deeply into the glycosylated region, eventually reaching the lipid core. The syringol molecule remains in the outer leaflet for approximately 50 ns prior to a rapid exchange to the inner leaflet, where it remains for approximately 100 ns before briefly entering into solution and completing a full membrane transit event (Supplementary Animation 1).

The rarity for full transits in our dataset supports the hypothesis that OMs pose a larger barrier to permeation than IMs for some compounds. In prior IM studies, phenol, guaiacol, and syringol readily translocated between leaflets in 500 ns equilibrium simulations (15), with multiple translocation events observed. Leaflet exchanges are also observed in the current membrane models, with the same three molecules readily crossing from the inner, unglycosylated leaflet into the opposing leaflet and back again (Figs. 2, S1, and S2). The missing component required for permeation are crossing events across the glycosylated region within the LPS-filled outer leaflet. While our dataset only contains a single permeation event, its existence within 100 microseconds of aggregated equilibrium sampling across all compounds suggests that the permeation rate across the OM may be fast on a biological timescale.

**Figure 3:**
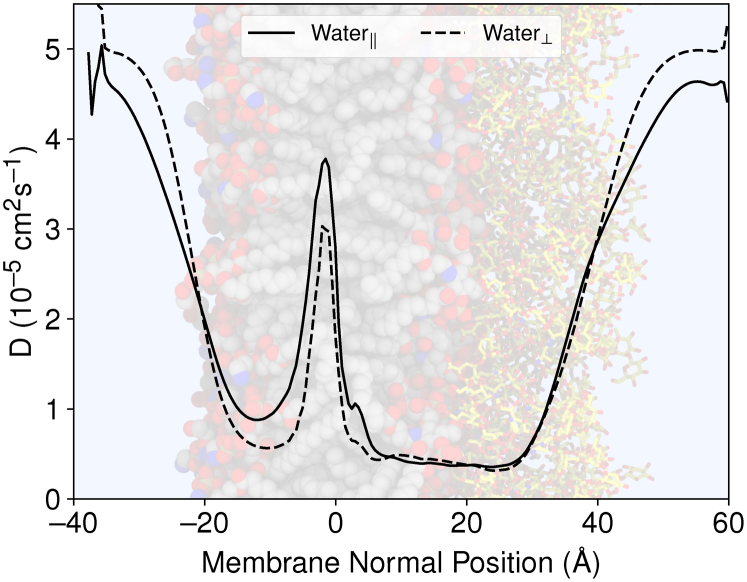
**Position-dependent water diffusion coefficients both parallel to (Water**_**k**_**) and perpendicular to (Water**_**⊥**_**) the membrane normal axis.** For context, the *black curves* indicating the diffusion coefficient are overlaid on a molecular graphics representation for the glycosylated membrane following the color scheme from Figure 10.

Mechanistically, slow permeation across the LPS leaflet may be related to the slow diffusion processes observed in that leaflet during the equilibrium trajectories. Animation S1 already hints that the dynamics within LPS leaflet are slower than in the phospholipid leaflet. Water diffusion parallel and perpendicular to the membrane normal axis is slower within the LPS leaflet than within the phospholipid leaflet (Fig. 3). Slower water diffusion within the LPS leaflet is not limited to the LPS glycosylations but also extends to membrane depths where LPS acyl tails predominate. Mechanistically, the dense glycosylations may order and trap water or potentially other similar small molecules. In a different measure of lipid dynamics, the LPS leaflet in our OM model diffuses nearly 100 times more slowly than the phospholipid leaflet (Table 1), in line with prior computational estimates for the independent OM leaflets (23, 24, 25). The slow lateral lipid diffusion generally is caused by the increased mass for LPS molecules and the abundant polar interactions within the LPS glycosylations. The slow diffusion processes caused by abundant interactions up and down the LPS chain slow all dynamics in the LPS leaflet, retarding membrane crossing.

**Table 1:** Lateral diffusion coefficients for LPS and phospholipid leaflets in our OM model

Leaflet	*Dlateral*
LPS	5*.*6 ± 0*.*3 × 10^−10^ cm^2^s^−1^
Phospholipid	4*.*9 ± 0*.*2 10^−8^cm^2^s^−1^

**Figure 4:**
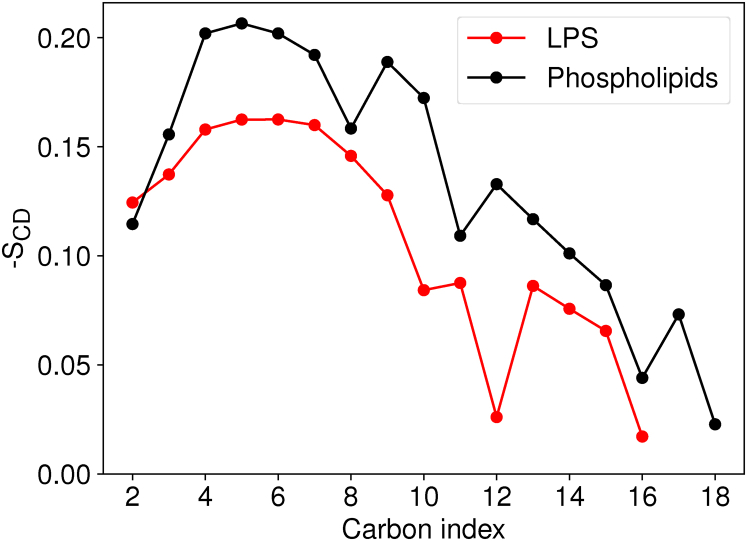
**Mean acyl tail order parameter (-S**_***CD***_**) computed for all acyl tails in the LPS (*red*) and phospholipid (*black*) leaflet.**$-S_{CD}=-<3cos^{2}\left(\theta_{CH}\right)-1>/2$ where *θ_CH_* is the angle between a C-H bond vector from the trajectory and the membrane-normal axis (60). As the number of samples behind each datapoint is so high across all 16.8*μ*s of equilibrium simulation, the SEM for each acyl tail order parameter does not rise outside the drawn point. LPS, lipopolysaccharide.

Notably, the slow dynamics in the LPS leaflet does not imply that the LPS leaflet is more ordered. Comparing between LPS and phospholipid tails within our model membrane indicates that the LPS acyl tails have a lower average order parameter at the equivalent carbon positions than their phospholipid counterparts (Fig. 4), indicating greater conformational variability in the LPS acyl tail than in the phospholipid tails. While prior LPS simulations did not explicitly compare acyl tail ordering with phospholipid acyl tails (26, 27), the magnitude and trends for -S_*CD*_ are consistent with prior results both for LPS (26, 27) and phospholipids (28). As we are averaging over multiple different acyl chains with a mixed lipid composition, the order parameter reduction near common unsaturation sites is less evident in Figure 4 than is typical from homogeneous lipid compositions that study each acyl tail independently.

### Predicting permeation coefficients

While equilibrium trajectory analysis serves to provide mechanistic insight into LRC translocation through bacterial OMs, the equilibrium trajectories alone are insufficient to compute accurate free energy profiles or kinetics for OM crossing. Without an applied biasing potential, the LRCs extensively and unproductively sample the reaction coordinate near membrane lipid tails where their interactions are most favorable. Through biased simulation, we are able to sample evenly across the bilayer translocation reaction coordinate, collecting statistics on rarely visited transition regions critical to determining the kinetics for OM permeation events.

**Figure 5:**
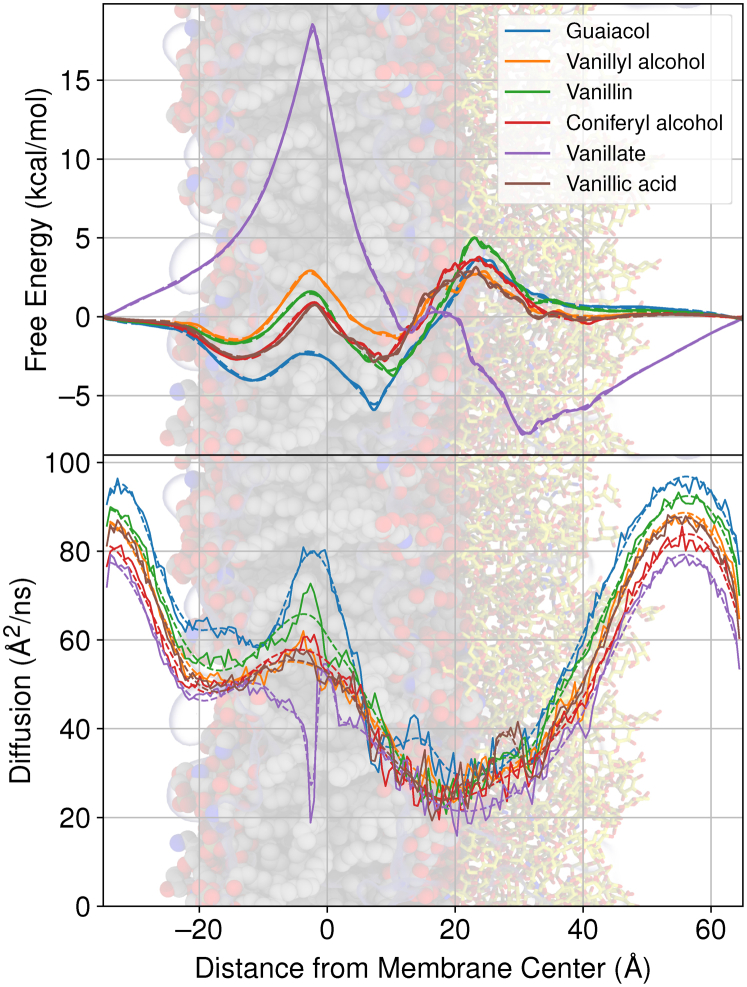
**Free energy (*top*) and diffusivity (*bottom*) profiles for selected G-type lignin monomers included in our test set** (Fig. 1). Each compound has two lines associated with the compound, a *solid line* reflecting the instantaneous best estimate for the quantity of interest and a *dashed line* indicating the spline fit used to numerically integrate Equation 3 to tabulate permeability coefficients in Table S1. For context, the plots are underlaid with a molecular representation for the glycosylated membrane, following the color scheme from Figure 10. Free energy and diffusivity profiles for other LRCs are provided in Figs. S42–S51.

In the inhomogeneous solubility diffusion model (Equation 3) (29, 30), the permeability coefficient depends only on the free energy and diffusivity profiles across a region of interest. The two essential profiles for selected G-type lignin monomers from our larger LRC set are provided in Figure 5 and indicate substantial changes in permeability depending on the LRC. The highest free energy barrier overall is observed for vanillate near the membrane midplane, reflecting the energetic cost for dehydrating the vanillate anion. If the carboxylate anion were to protonate, as in vanillic acid, the energetic penalty for crossing the membrane is substantially reduced. For many of the other LRCs tested, the largest barrier to permeation was observed at the interface between the lipid and polysaccharide portions for the LPS leaflet. At this interface, typical free energies for uncharged species were between 2-6 kcal mol^−1^ relative to solution (Figs. 5 and S42–S51), representing a significant but not insurmountable barrier to permeation.

The free energy for uncharged LRCs was typically negative within the membrane core relative to solution, suggesting favorable partitioning for these small molecules into the membrane and rapid, low-barrier exchanges between leaflets. The free energy profiles also suggest that the preferred insertion depth for an LRC was leaflet dependent, with the LPS leaflet demonstrating a preferred insertion depth nearer to the membrane midplane than the phospholipid leaflet. The shorter acyl tail lengths and lower lipid ordering for LPS membrane components means that carbonyl-adjacent regions for the membrane are closer to the midplane for an LPS-containing leaflet than for our phospholipid model. Likewise, other equivalent condition regions within the membrane core exist and are shifted with respect to the membrane midplane. Another common feature for the LRC free energy profiles are barriers near the transition between the LPS and phospholipid leaflets. We ascribe the offset between the membrane center and the free energy maxima to the dynamical differences between the LPS and phospholipid leaflets, as the membrane center was defined based on the center of mass for the acyl tail termini.

The diffusion coefficient profiles are similarly asymmetric in a leaflet-dependent manner. The diffusivity along the membrane normal within the phospholipid leaflet compares favorably with prior measures for diffusion coefficients along membrane transfer axes (15). For phospholipid membranes, the aqueous diffusion coefficients for LRCs are near 80Å^2^/ns, exhibiting a minima near the membrane/water interface before increasing LRC diffusion near the membrane center. The diffusive behavior within phospholipid leaflets, including diffusion minima near interfaces, have been noted previously for gas diffusion within membranes (31, 32), although alternative analysis methods yield alternative diffusivity profiles (30, 33). Qualitatively, the trends are similar for the LPS leaflet. In the LPS leaflet, the diffusion coefficient is high in aqueous solution and somewhat lower at the membrane center. The slowest diffusion occurs near the membrane-glycosylation interface as the LRCs transition between lipophilic and hydrophobic regions. The diffusion at the membrane-glycosylation interface is approximately half the rate as what was observed in the membrane-water interface for the phospholipid leaflet (Fig. 5). The diffusive reduction in LPS is comparable in magnitude to the water diffusion parallel to the membrane normal axis presented in Figure 3.

With the free energy and diffusivity profiles in hand, we are able to compute partitioning and permeability coefficients for the diverse LRCs in our simulation set (Tables 2 and S1) based on Equations 3 and 4. The partition coefficients are nearly uniformly positive, indicating that the LRCs partition into the membrane, with only a few charged molecules preferring solution (Table S1). The permeability coefficients vary by up to 13 orders of magnitude, with charged compounds such as vanillate demonstrating the slowest permeation. For uncharged species, particularly for common compounds enumerated in Table 2, permeability was often limited by permeation through the glycosylation attached to the LPS leaflet (*Pm*_*g*_), consistent with the identified regions of high free energy and low diffusion (Fig. 5).

**Table 2:** Partition (P) and Permeability (Pm) coefficients for G-type LRCs shown in Figure 5 in the OM mimic

Compound name	logP	log*Pm*(*cms*^−1^)	log*Pm*_*u*_ (*cms*^−1^)	log*Pm*_*c*_ (*cms*^−1^)	log*Pm*_*g*_ (*cms*^−1^)
Guaiacol	4.0	-0.8	-1.9	-0.3	-4.8
Vanillyl alcohol	1.0	-0.4	0.8	-0.9	-1.3
Vanillin	2.5	-1.6	-0.5	-1.4	-4.1
Coniferyl alcohol	1.9	-1.0	-0.0	-0.4	-2.9
Vanillate	1.3	-10.7	-0.9	-12.0	1.0

The permeability coefficient of crossing the entire membrane, going from aqueous solution to aqueous solution, is decomposed into a crossing permeability (Pm_*c*_) and extraction permeabilities into solution through the glycosylated (Pm_*g*_) and unglycosylated (Pm_*u*_) sides. The decomposition is done by adjusting the integrated bounds in Equation 3 to integrate between free energy minima found near the lipid-water and lipid-glycosylation interfaces. This is described by Equation 5 in the methods. The same information for all tested compounds is available in Table S1. Typical uncertainties for the tabulated quantities are 0.2 log units.

## Discussion

Based exclusively on the results presented, the initial hypothesis that the LPS layer in a *P. putida* OM reduces LRC permeability appears plausible. Spontaneous permeation is rare within our simulation set (Fig. 2), and the glycosylations frequently present the largest barrier to permeation (Fig. 5 and Table 2). To demonstrate what practical impact the OM might have on LRC uptake on *P. putida*, we take the opportunity here to place the OM permeation coefficients into their biological context.

### Double membrane permeability and LRC chemistry

**Figure 6:**
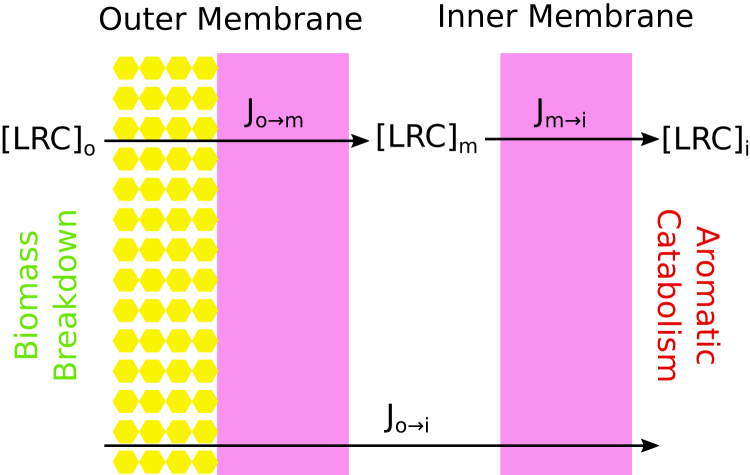
**Schematic for the simplified membrane transport processes through both the OM and IM in *P. putida*, highlighting the three primary aqueous compartments featuring LRC concentrations.** Biomass breakdown increases LRC concentration outside the cell ([LRC_*o*_]), while aromatic catabolic processes inside the cell reduce the LRC concentration ([LRC_*i*_]). This establishes a concentration gradient that approaches steady state when the fluxes across the individual membranes (*J*^*o*→*m*^ and *J*^*m*→*i*^). In this schematic, the hydrophobic membrane regions are represented by *pink rectangles*, while the OM LPS glycosylations are represented by *yellow hexagons*. IM, inner membrane; OM, outer membrane; LRC, lignin related compound; LPS, lipopolysaccharide.

Our model for LRC uptake in *P. putida* is quite simple, consisting of a LRC source external to the cell derived from biomass breakdown, an LRC sink representing internal metabolism, and the *P. putida* OM and IM (Fig. 6). The modeled passive transport system reaches a steady state where the molecular flux into the cell matches consumption by cellular metabolism. At steady state, *J*_*o*→*m*_ = *J*_*m*→*i*_ = *J*_*o*→*i*_, where the effective permeability coefficient for the total process (Pm_*t*_) can be calculated from the permeability across the OM (Pm_*o*_) and IM (Pm_*i*_).

(1) $$Pm_{t}=\frac{Pm_{o}Pm_{i}}{Pm_{o}+Pm_{i}}$$

Based on the structure of Equation 1, Pm_*t*_ is approximately half the permeation coefficient for either membrane when Pm_*o*_ and Pm_*i*_ are similar in magnitude. Otherwise, Pm_*t*_ will be limited by the smaller of Pm_*o*_ and Pm_*i*_.

**Figure 7:**
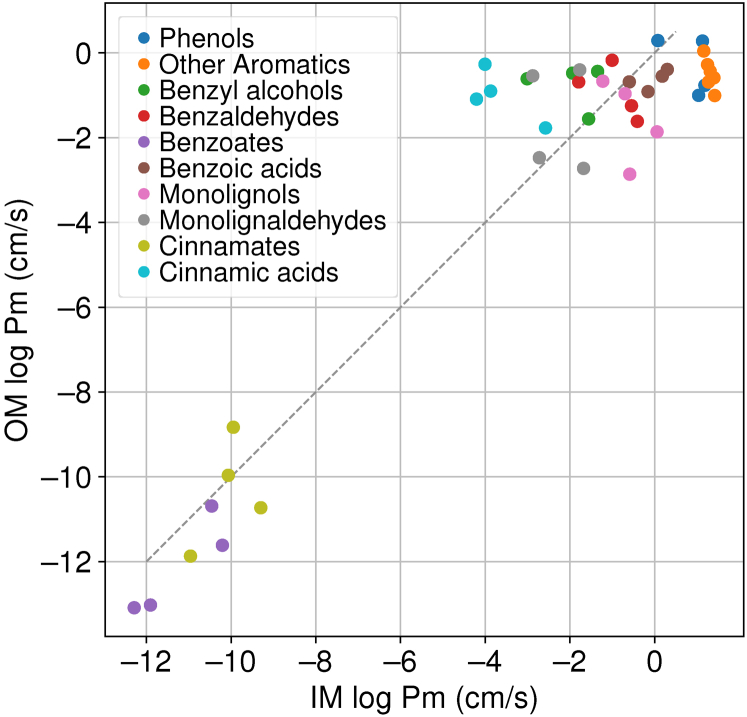
**Outer-versus inner-membrane permeability for compounds within our test set (**Fig. 1**) where IM permeability had been previously calculated** (15). To guide the eye, we have added a *dashed line* to indicate where OM Pm = IM Pm. Below this line, Pm_*o*_*<* Pm_*i*_, indicating that the OM is rate limiting. The converse is true above the line. IM, inner membrane; OM, outer membrane.

Thus, when looking at the larger passive transport process, the results from Tables 2 and S1 need to be compared with prior results for the IM (15). From Figure 7, we observe variation in relative permeation coefficients dependent on LRC chemistries. LRCs with additional oxygenation sites, such as cinnamic acids, are limited by the IM permeability rather than the OM permeability, which can be up to 1000 times larger. More lipophilic LRCs may have IM permeability coefficients 100 times larger than the OM permeability coefficient. These lipophilic LRCs include phenols found in aqueous waste streams from catalytic fast pyrolysis (34). Other compounds have computed permeability coefficients that are comparable in magnitude. Thus, even though the largest barrier to crossing the OM is typically within the LPS glycosylation region, the overall impact on passive permeation coefficient across both bilayers is relatively low. The permeation barrier provided by the LPS glycosylation may reduce Pm_*t*_ by 3 orders of magnitude. By comparison, changing LRC chemistry to include a negative charge reduces Pm_*t*_ by approximately 10 orders of magnitude, a far more significant change with more far-reaching biological consequences.

**Figure 8:**
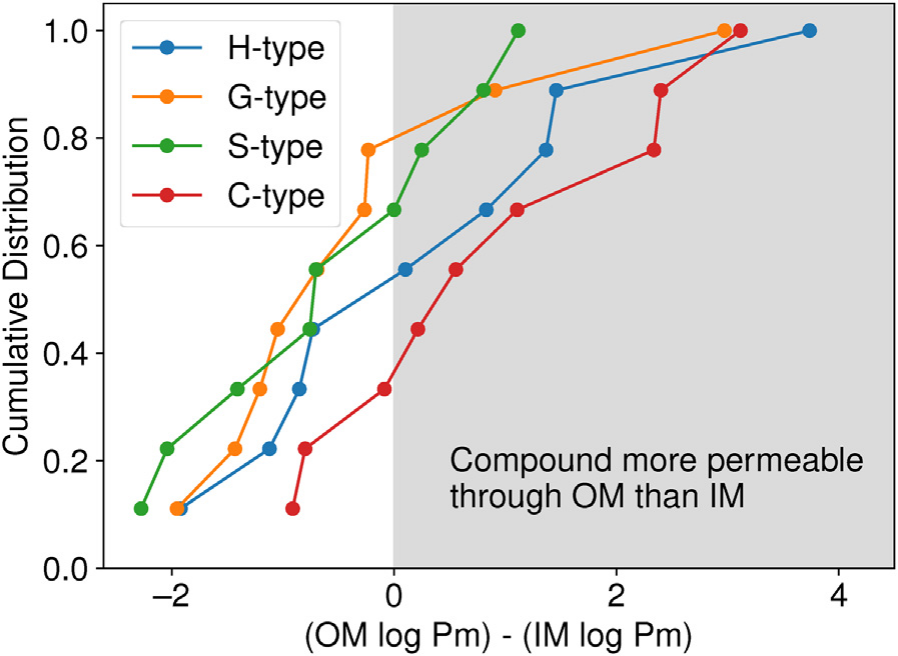
**Replotting the data in**Figure 7**by the type of lignin monomer as a cumulative distribution function, determining whether the small molecules are more likely to permeate through the OM or the IM.** IM, inner membrane; OM, outer membrane; LRC, lignin related compound; LPS, lipopolysaccharide.

By regrouping the information from Figure 7 by the LRC type from Figure 1, we can evaluate how hydroxylation and methoxylation for LRC compounds change permeability (Fig. 8). From Figure 8, we find that methoxylated S- and G-type LRCs have distributions shifted to the left in Figure 8 compared with H- and C-type LRCs that are only hydroxylated. The shifted distribution indicates that the methoxy-substituted LRCs are less permeable across the OM than their hydroxylated counterparts. Hydroxyl groups can act as hydrogen bonding donors and acceptors, unlike methoxy groups, which can only act as hydrogen bonding acceptors. Within the LPS region, we find that hydroxyl groups form significantly more hydrogen bonds per functional group (Fig. S52). The favorable interactions with the LPS region lower the permeation barrier for hydroxylated LRCs. Thus, one could hypothesize that hydroxylated LRCs may be catabolized more quickly by *P. putida*.

### Practical flux calculations

With a permeability coefficient in hand for the combined OM and IM system based on Equation 1, we can calculate potential fluxes based on the permeability coefficient, the area for the permeating surface A, and the concentration gradient across the dividing surface.

(2) $$J_{o\to i}{=\text{Pm}}_{t}{A(\left[\text{LRC}\right]}_{o}{-\left[\text{LRC}\right]}_{i})$$

The key surface area and concentration gradient parameters within Equation 2 can be estimated from experimental sources. High mM titers for phenolic LRCs have been observed in prior microbial engineering efforts to produce phenols (35, 36) and would represent an upper bound for the concentration difference within Equation 2 *P. putida* could tolerate. The membrane surface area for *P. putida* can be estimated from micrographs showing a rod-like shape with long axis of approximately 1 *μ*m and a 0.4 *μ*m diameter (16), yielding 1.25 *μ*m^2^. Assuming a 1 mM concentration difference, the net inward flux for phenol predicted by Equation 2 is 25 fmol/s (1*.*5 × 10^10^ molecules/s), limited by Pm_*o*_=2 cm/s. While the OM limits flux and reduces permeation 10-fold compared with prior IM simulations (15), the potential permeation rate remains very high in this example.

Many LRCs tested will exhibit similar behavior, as their permeabilities are broadly similar to that of phenol. Among the uncharged LRCs, sinapyl alcohol has the smallest permeability coefficient, with Pm= 10^−2*.*9^ cm/s, around 1600 times less permeable than the phenol example above, yielding permeation rates on the order of 10^7^ molecules/s. With this high potential flux, the actual flux is likely controlled by the steady state rate of LRC synthesis or catabolism. Enzymes involved in aromatic demethylation turn over few times a second (37, 38), and lignin acetylation enzymes turn over in tens of seconds (39). Slow lignin metabolism implies that passive flux across the OM is not rate limiting, even in the absence of specific transport proteins. Instead, the concentration gradient implied by Equation 2 for uncharged LRCs would shrink such that the flux matches metabolic processes.

For charged LRCs, permeability is substantially slower. If we replace phenol from our example above with *p*-hydroxy-benzoate, the permeation rate would slow to a few molecules per hour. For such a small flux, ingress and egress control by transport proteins is highly likely, tempered by the possibility that many permeation events for carboxylates may occur when the carboxylate protonates to form the neutral acid. The acids demonstrate permeability coefficients that are significantly faster and thus may represent the major species that passively permeates, particularly in acidic conditions.

### Model limitations

While the main message that OM permeability is actually quite fast for LRCs is clear, there are potential areas of concern for our models. The first is how well the inhomogeneous solubility diffusion model and the underlying simulation methods and force fields recapitulate reality. Indeed, systematic surveys suggest that molecular simulation can overestimate permeability by an order of magnitude (30). In our view, a modest systematic bias towards accelerated permeability is acceptable, as the potential passive fluxes are sufficiently large that overestimating these by a factor of 10 does not materially change whether permeation or metabolism limits the steady state flux through the system.

However, a larger area of uncertainty arises from our choice for a relatively thin LPS layer. Removing the O-antigen entirely was necessary from a computational perspective to keep the simulation sizes tractable for a large set of LRCs. Consequently, we may overestimate permeability through the LPS layer, as additional glycosylation may offer greater resistance to permeation. If we make the assumption that the free energy and diffusivity across the additional O-antigen thickness can be taken from point where the free energy reaches a maximum within the LPS (Equation 6), we find that the O-antigen thickness required to reduce permeability tenfold is between 2.8 and 14.2 nm for uncharged LRCs. Since additional thickness would only linearly add resistance to permeation (29, 30), it is likely infeasible for realistic O-antigen lengths to reduce OM permeability by more than 10 to 100 times unless we underestimate the free energy barrier in our current model construction.

**Figure 9:**
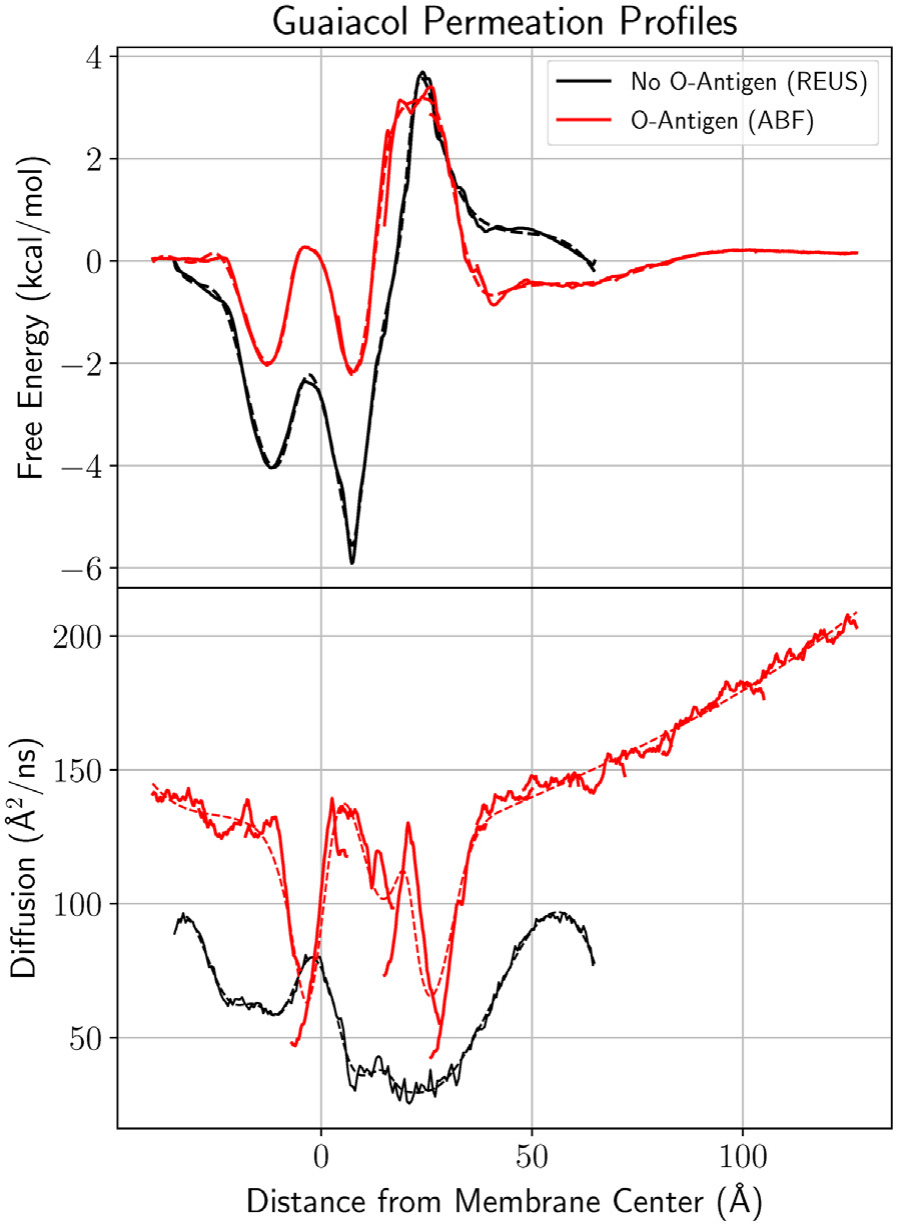
**Free energy and diffusivity profile comparison for guaiacol.** Two profiles are shown here, one where the membrane lacked an O-antigen and the profiles were computed *via* REUS calculations (*black line*, used throughout the text) and another profile set computed with a membrane containing an O-antigen, where the profile was computed *via* ABF (*red line*). *Dashed lines* represent the spline fits used to integrate Equation 3. ABF, adaptive biasing force; REUS, replica exchange umbrella sampling.

To test this hypothesis, we directly compare free energy and diffusivity profiles determined through adaptive biasing force (ABF) calculations for guaiacol in a membrane where the O-antigen is present to the original replica exchange umbrella sampling (REUS) simulations that lacked the O-antigen (Fig. 9). Overall, the free energy profile has a similar structure, with minima near the acyl tail carbonyls and a maxima where the LPS leaflet transitions from a lipophilic to a hydrophilic environment. Crucially, the barrier within the free energy profile has a similar maximum and returns to zero in the glycosylation region both with and without O-antigen present. Prior studies that calculate free energy barriers across LPS membranes arrive at similar small molecule free energy profiles (40), suggesting that indeed the interface between the hydrophobic and hydrophilic regions within LPS are the primary barrier to permeation for nonpolar compounds. The extended sampling within the ABF calculation appears to have broadly improved the symmetry for the profile within the membrane.

The diffusivity profiles are substantially different between the two methodologies, which have been noted previously when comparing REUS and ABF results (30). We also note that our ABF sampling strategy created discontinuities within the diffusivity profile for the O-antigen membrane, which we attempt to correct *via* a spline fit (Fig. 9). In truth, the diffusivity matters very little in the overall permeability picture. Remembering that the computed guaiacol permeability coefficient is 10^−0*.*76^ cm/s when the O-antigen is absent (rounds to −0.8 in Table 2), we integrate Equation 3 to estimate permeability when the O-antigen is present. The equivalent coefficients computed from the ABF results are either 10^−0*.*81^ cm/s (using REUS diffusivity and ABF free energy) or 10^−0*.*40^ cm/s (using ABF for both diffusivity and free energy). Based on this limited evidence, adding or removing the O-antigen on LPS does not materially change the permeability for the OM as a whole for LRCs.

While the permeability through the OM and IM for uncharged LRCs is significant, the inherent assumption behind Equation 2 is that the peptidoglycan layer sandwiched between the OM and IM contributes little resistance to LRC permeability. While the peptidoglycan layer in *P. putida* is in an aqueous phase, interactions between the dense peptidoglycan mesh (41) and the LRCs may impede permeation. Strong glycan–lignin interactions have been observed in prior simulations (42, 43). However, the space between the OM and IM is restricted, and further study is required to determine how peptidoglycan and lignin interact.

## Conclusion

Within a biorefinery context, where substrate concentrations are at the limit of what the organism can tolerate and metabolize, passive permeation for most LRCs through the OM will likely not be rate-limiting to bioconversion. Permeability coefficients for the OM are broadly similar to the IM counterparts, with the OM rate limiting for the most lipophilic LRCs while the IM is rate limiting for hydrophilic LRCs. The differences between the OM and the IM were largely attributable to the LPS leaflet found on the OM. Diffusion with the LPS layer is substantially slower than elsewhere in the OM, as interactions with LRCs and solution are made and broken with the LPS glycosylations. Charged substrates, however, permeate exceptionally slowly through the OM, limited by the unfavorable energetics for desolvating an ion as it passes through lipophilic membrane regions. Thus, we expect transport proteins to be intimately involved in carboxylate uptake by *P. putida*, while other LRCs permeate freely through a passive mechanism, depending on metabolic processes to generate the concentration gradient needed to drive flux across the OM.

From a process design perspective, the ability for many LRCs to permeate passively eliminates some constraints on metabolic engineering approaches in other systems. For instance, sucrose synthesized photosynthetically depends on the overexpression of a sucrose–proton symporter for export at high titers (44), as sucrose cannot passively permeate bacterial membranes at an appreciable rate. While the final product from metabolically funneling LRCs may similarly require a specific transport protein for harvesting product, the inputs for bioconversion can be sourced without substantial engineering effort invested in importing the diverse chemistries represented by the range of LRCs tested. Thus, bacteria with flexible metabolisms that can process a wide range of LRCs and waste aromatics from other sources represent excellent candidates for future metabolic funneling without engineering specific transport proteins.

## Experimental procedures

**Figure 10:**
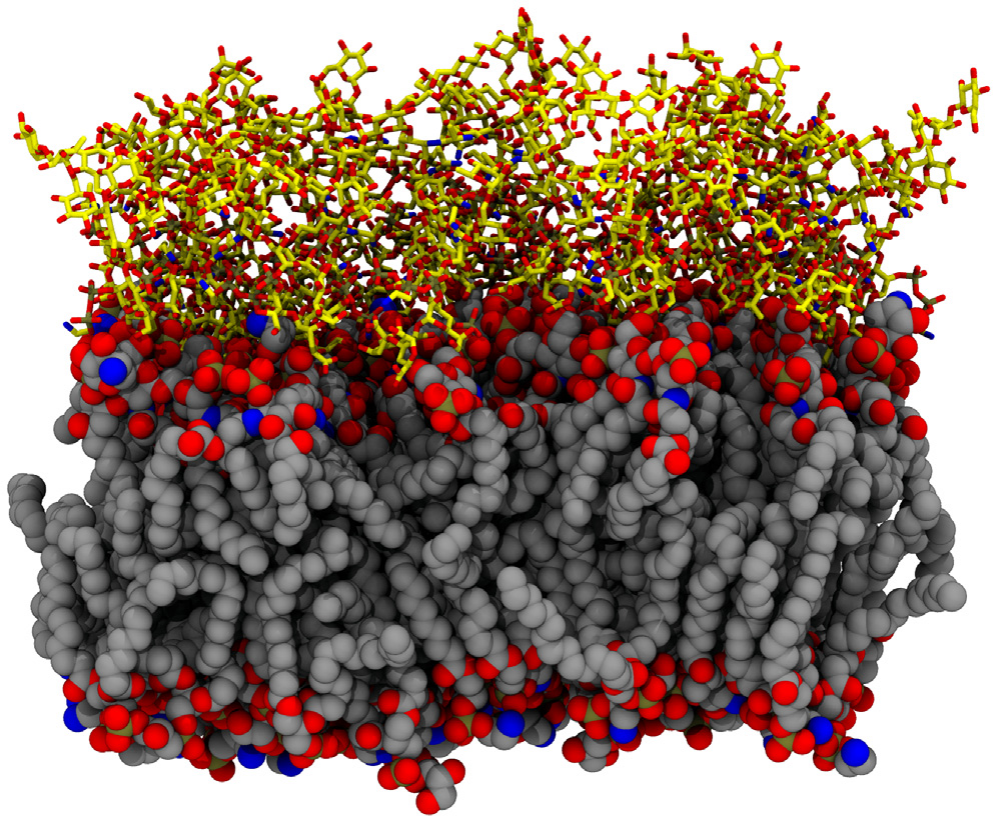
**Membrane construction snapshot.** The heavy atoms for the lipid headgroups and acyl tails are shown as *spheres*, utilizing *gray* carbons to contrast with the *yellow* carbons featured in the glycosylations attached to the outer leaflet, represented with *sticks*. Other atoms have a consistent color scheme, with *blue* representing nitrogen, *red* representing oxygen, and *brown* representing phosphorus. Hydrogen and solvating water molecules, while present during simulation, are omitted for visual clarity.

The general procedure for computing OM permeability closely follows prior permeation studies in the group for small molecules across biological membranes (15, 45, 46). First, we carry out equilibrium MD simulations for a set of 42 LRCs (Fig. 1) in the context of a model bacterial OM from *P. putida* (Fig. 10), checking for spontaneous permeation as was observed for other small molecules through biological membranes (45, 46, 47). The equilibrium MD complements REUS simulations that allow for permeability to be calculated through the inhomogeneous solubility-diffusion model (29, 30, 45, 48, 49), even if no spontaneous permeation events are observed and the timescale for permeation is unknown. The inputs and selected outputs are available on Zenodo.

The chosen LRCs for this study (Fig. 1) were drawn from a subset of the LRCs where permeation across a model *P. putida* IM was calculated (15), as the larger simulation systems required to capture a glycosylated OM made repeating the same suite of compounds cost prohibitive. The 42 selected LRCs in Figure 1 reflect the diverse chemistries seen in lignin but are tailored to focus on monomeric products that could result from native lignin polymer deconstruction (50, 51). Other simple aromatics tested reflect species that might be encountered by a bacterial host in a mixed waste stream (5, 52, 53).

### Membrane construction

The OM of *P. putida* is composed of diverse LPSs). Lipidomics and glycomics have not elucidated the distribution of potential LPS species in enough detail for molecular modeling. Thus, our OM model uses 30 diverse LPS molecules modeled on what is found in *Pseudomonas aeruginosa* and makes specific structural choices to support our research objectives. The 30 LPS molecules for the OM were constructed through CHARMM-GUI (20, 27, 54, 55), six for each of the five available lipid A structures, using core 1B to represent the glycosylation pattern. Unlike other recent studies for the OM in *P. aeruginosa* (22), no O-antigen glycosylations were added to the LPS molecules. From a computational perspective, a minimal O-antigen would add at least another 4 nm of membrane thickness (22) for LRCs to traverse, and it is not immediately clear what the correct O-antigen length would be for *P. putida*. We estimate that additional glycosylation from a standard O-antigen would further increase the system size by around 40%. The increased system size decreases performance for individual simulations, as well as increasing the number of umbrellas that would be needed to span the bilayer when computing permeability. Thus, we estimate that adding in the O-antigen would effectively double the total computational cost for determining permeability. We approximate the impact for the O-antigen by assuming that the highest barrier we compute across the glycans is maintained for additional O-antigen thickness.

The inner leaflet was also generated using CHARMM-GUI (27, 54, 55). The composition for the inner leaflet matches the composition for IM models for *P. putida* used previously (15), with an approximate 2:1 PE:PG headgroup ratio to match *P. putida* lipidomics studies (56). The final number of lipids in the inner leaflet (75) was chosen to match the membrane surface area for the LPS outer leaflet. The membrane is solvated and ionized within CHARMM-GUI, with 114 Ca^2+^, 114 K^+^, and 22 Cl^–^ ions. Calcium ions are chosen for ionization here based on the structural evidence for their importance to maintain OM stability (57, 58). When the membrane geometry is complemented with small molecules, each molecular system is approximately 55,000 atoms in size, with equilibrated dimensions of approximately 75 × 75 × 90 Å.

### Equilibrium simulation

To evaluate spontaneous permeability for each LRC considered (Fig. 1), 10 LRC molecules were added to the previously generated membrane (Fig. 10) to create independent simulation systems for each LRC. All 42 systems were simulated for 400 ns using NAMD 2.13 (59). The systems were simulated using the CHARMM36 force field, including terms for lipids (60), carbohydrates (61, 62, 63), and lignin (64). The CHARMM36 General force field (65) was used for small organic molecules not covered by the lignin forcefield (Other Aromatics category in Fig. 1). Aqueous solution was represented with the TIP3 water model (66) and ion parameters from Beglov and Roux (67).

Other simulation parameters shared with the subsequent REUS calculations include a 12 Å nonbonded cutoff with switching applied past 10 Å and long-range electrostatics treatment using the particle mesh Ewald method (68, 69) with 1.2 Å grid spacing. Semi-isotropic pressure coupling was maintained *via* the Langevin piston method (70, 71) to a pressure of 1 atm, with periodic cell dimensions along the membrane normal axis decoupled from growth along the membrane parallel axes. A Langevin thermostat (72) using *γ*=1 ps^−1^ maintained the system temperature at 310K. Each simulation timestep was 2 fs, enabled by using the SETTLE algorithm (73) to fix bond lengths to hydrogen.

### Replica exchange umbrella sampling

To obtain a free energy profile and local diffusivity for our selected LRCs along the reaction coordinate representing passive membrane transit across the OM, we carried out a set of REUS (74) (also called Hamiltonian replica exchange (75), window exchange umbrella sampling (76), or bias exchange umbrella sampling (77)) simulations. Similar to conventional umbrella sampling, REUS samples high energy regions of the reaction coordinate range by applying a harmonic bias (umbrella) to the system, forcing uniform sampling across a reaction coordinate (78), rather than just the low energy regions of the free energy surface as would occur without an applied bias. Given known biases, unbiased free energy surfaces can be estimated from these biased simulations using well-established techniques (79, 80).

For our system, the chosen reaction coordinate reflects permeation for the center of mass for a single LRC molecule across the membrane, going from -35Å on the inner leaflet side to 65Å on the outer leaflet side. As each umbrella restricts the sampling to a small fraction of the total reaction coordinate, a series of 180 independent umbrellas with 4 kcalmol^−1^ force constants were used to cover the entire reaction coordinate, yielding a 0.55 Å umbrella spacing. This spacing and force constant are consistent with what has worked previously to evaluate permeation in other systems (15, 30).

The primary advantage of REUS relative to conventional umbrella sampling is ensuring contiguous sampling, as the individual umbrella biases are exchanged periodically according to a Metropolis criterion (75, 74, 81), accelerating sampling along the chosen reaction coordinate. Adjacent windows were attempted to be exchanged with alternating neighbors every 1 ps, consistent with advice to exchange often during replica exchange simulations (82). To set up a diverse pool of starting configurations for each replica, initial configurations for single LRC molecules in an OM system are drawn at random from the computed equilibrium trajectories. For computing permeabilities, REUS achieves the same level of accuracy as conventional umbrella sampling with shorter simulation times (30), and based on past experience (15), each window was sampled for 40 ns.

### ABF calculation

**Figure 11:**
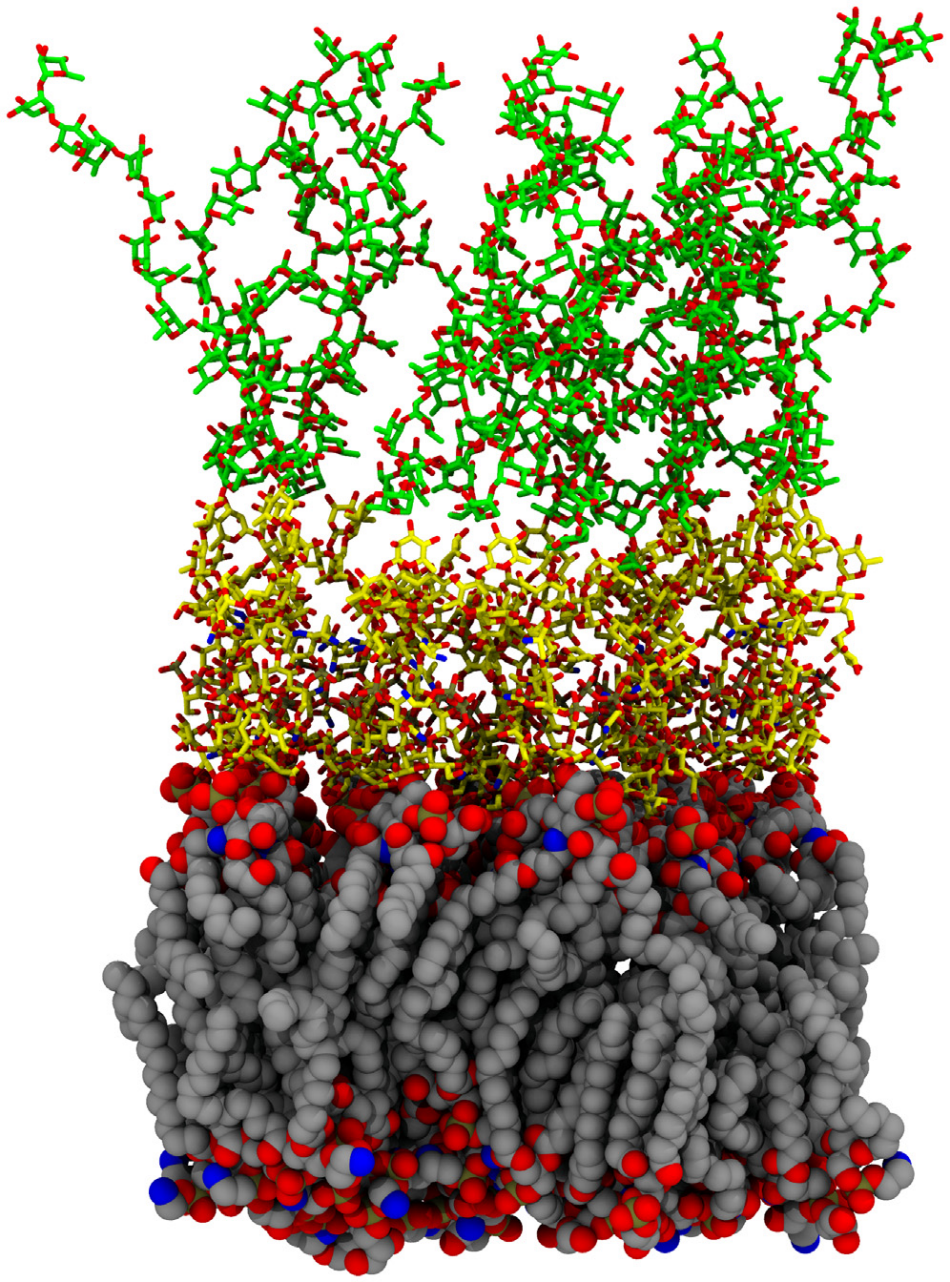
**Initial snapshot for the membrane system with the O-antigen added.** The representations used are analogous to Figure 10, with the 15 additional rhamnose monomers on each LPS head shown with *green carbons*, while the glycosylated core retains the *yellow carbons* from Figure 10. LPS, lipopolysaccharide.

To assess the impact for the O-antigen on LRC permeation, we determined the local free energy and diffusivity profiles using ABF (83, 84) simulations. The membrane setup matches the membrane composition described previously but adds O-antigen consistent with the *P. aeruginosa* default composition from the CHARMM-GUI membrane builder (55). This adds 15 rhamnose monomers to the membrane, increasing the height for the membrane considerably (Fig. 11). Once solvated and ionized as noted above, the initial system is 60 × 60 × 150 Å.

To sample the membrane normal dimension efficiently, the full membrane normal span was split into 15 independently sampled reaction coordinates. Each collective variable defining the space was 13 Å wide with 2 Å overlap between adjacent collective variables, spanning from -40 Å below the membrane midplane to 127 Å above the membrane midplane. A single guaiacol molecule sampled each bounded reaction coordinate, such that 15 guaiacol molecules in total were present within the membrane-water system. To further increase sampling, eight ABF simulations were performed simultaneously and shared biasing information through the multiple walker framework every 5000 steps (85) within NAMD 2.14 (59). Thus, the eight individual 378 ns simulations performed represent over 45*μ*s of sampling for the membrane normal reaction coordinate, substantially longer than the 7.2*μ*s needed for a converged PMF with REUS.

### Analysis

The analysis procedures largely follow our prior work on permeability across biological membranes (15, 45, 46). Python scripts combining VMD (86), numpy (87, 88), and scipy quantified simulation trajectory observables. Molecular visualizations were rendered using VMD (86) with its built-in GPU-accelerated OptiX raytracer (89), and dataplots were made with matplotlib (90). From the equilibrium trajectories, we track the location of individual LRC molecules to identify complete crossing events, as well as membrane-depth dependent estimates for solvent diffusion. Diffusion for water and lipids within the equilibrium trajectories are determined *via* the Einstein relation (91) in either two dimensions for lateral diffusion or in one dimension for diffusion along the membrane normal axis. We do so by tracking displacements at 20 ps intervals and binning the resulting displacements based on position to estimate position-dependent water diffusion from equilibrium simulation. The carbon-deuterium order parameter measuring structure for the acyl tails (-S_*CD*_) through NMR is approximated by the carbon-hydrogen order parameter used in parameterizing the lipid force field (60). The REUS calculations are the basis for calculating the permeability and partitioning coefficients, based on free energy and diffusivity profiles derived from the REUS trajectories.

The free energies G and diffusivities D along a reaction coordinate *ξ* bounded by upper and lower boundaries *ξ*_*u*_ and *ξ*_*l*_ are converted into permeability coefficients (Pm) through the inhomogeneous solubility-diffusion model (29, 30) originally postulated by Diamond and Katz (92).

(3) $$Pm={\left[\int_{\xi_{l}}^{\xi_{u}}\frac{\exp\left(\Delta G\left(\xi \right)\beta \right)}{D\left(\xi \right)}d\xi \right]}^{-1}$$

We further break down the permeability coefficient into three subcomponents representing a compound crossing the hydrophobic region (Pm_*c*_), exiting the membrane across the glycosylations (Pm_*g*_), and exiting the membrane across the unglycosylated side (Pm_*u*_) by splitting the integration range within Equation 3 into three parts. The integration boundaries splitting the integral to create the three subcomponents were determined by the free energy minima in the range (-22,-5) for the unglycosylated side boundary and the range (5, 22) on the glycosylated side boundary. The partition coefficient depends solely on the free energy difference between aqueous solution and the membrane.

(4) $$\log\;P=\frac{G_{aq}-G_{membrane}}{RT\;\ln\;10}=\frac{\Delta G_{partition}}{RT\;\ln\;10}$$

In this form, we use the simple approximation that G_*membrane*_ is the lower free energy minimum at the integration boundaries determined for permeability. The free energy reference state while integrating Equation 3 to obtain the subcomponents is chosen to be the minimum free energy. As a result, the permeability coefficient for traversing the entire membrane depends on the partition coefficient computed from Equation 4 to reset the free energy reference to capture the real kinetics for moving from solution to solution. In mathematical terms, this works out to be:

(5) $$\log\left[Pm\right]=\log\;P+\log\left[{\left(Pm_{u}^{-1}+Pm_{c}^{-1}+Pm_{g}^{-1}\right)}^{-1}\right]$$

To estimate the O-antigen thickness needed to decrease permeability by tenfold, we first find the position *ξ*_*max*_ where the free energy is maximized within the LPS layer. At this position, the integrand for Equation 3 ($\frac{\exp\left(\Delta G\left(\xi_{\max}\right)\beta \right)}{D\left(\xi_{\max}\right)}$) is maximized, which represents the incremental resistance to permeation (29, 30) as the membrane thickens due to longer/larger O-antigen bands. We solve for the thickness L needed to increase the resistivity *R* = *Pm*^−1^ ten-fold, which would reduce the permeability by an order of magnitude.

(6) $$10R=\frac{\exp\left(\Delta G\left(\xi_{\max}\right)\beta \right)}{D\left(\xi_{\max}\right)}L$$

The free energy profile from the REUS trajectories were calculated from a modified version of BayesWHAM (80), which uses Gibbs sampling of the known Dirichlet prior (93) to rapidly assess uncertainty. We use the free energy in solution as our zero point and force the equivalence for the free energy at the edges of our reaction coordinate by treating the reaction coordinate as periodic. The periodicity assumption has the largest impact on the calculated profiles for charged LRCs, namely the benzoates and cinnamates, as dehydrating ionic species during permeation can yield large sampling errors that are remedied in part by symmetrization (94, 95). Diffusivity profiles for Equation 3 were computed directly from the variance and autocorrelation time of the biased motion along the reaction coordinate (96), which is less sensitive to the harmonic restraint force than alternative calculation approaches (97) and is not influenced by concerns surrounding momentum removal (98). To compute auto-correlation times needed for the diffusivity calculation, we fit the autocorrelation function to an exponentially decaying function within the 1 ps intervals between exchanges. This has been previously identified to be a suitable method for estimating small molecule diffusivity from REUS simulations (45). The free energy profile from the ABF simulation was stitched together from the 15 independent reaction coordinates in numpy (87, 88). Diffusivity estimates directly from the ABF simulation were computed using DiffusionFusion, a C implementation for calculating position-dependent diffusivity from ABF calculations (99).

## Data availability

The reduced directory structure that includes analysis scripts, inputs, and selected raw outputs used for this publication is available from http://doi.org/10.5281/zenodo.5794252.

The complete directory structure is available upon request.

## Supporting information

The Supporting Information contains a pdf file with Table S1 reporting the partitioning and permeability coefficients for all 42 compounds. Figures S1–S41 supplement Figure 2. Figures S42–S51 report the free energy and diffusivity profiles for all 42 compounds, analogous to Figure 5. The free energy and diffusivity profiles are also tabulated numerically as an excel spreadsheet. Animation S1 shows the permeation of a single syringol molecule across the membrane in unbiased simulation, complementing Figure 2.

## Conflict of interest

The authors declare that they have no conflicts of interest with the contents of this article.

## Supporting information

Animation S1
