# Supplementary material for: Molecular simulation of lignin-related aromatic compound permeation through gram-negative bacterial outer membranes
Source: J Biol Chem. 2022 Oct 21;298(12):102627. doi: 10.1016/j.jbc.2022.102627 (PMC9720347; doi:10.1016/j.jbc.2022.102627)
Supplement: Supplemental data [file mmc2.pdf]

Supporting Information: Molecular simulation of lignin-related aromatic compound permeation through gram-negative bacterial outer membranes

Josh V. Vermaas,[*,†] Michael F. Crowley,[*,‖] and Gregg T. Beckham[*,‖]

†*Biosciences Center, National Renewable Energy Laboratory, 15013 Denver West Parkway, Golden, CO 80401*

‡*National Center for Computational Sciences, Oak Ridge National Laboratory, 1 Bethel Valley Road, Oak Ridge, TN 37830*

¶*MSU-DOE Plant Research Laboratory, Michigan State University, 612 Wilson Road, East Lansing, MI 48824*

§*Deparment of Biochemistry and Molecular Biology, Michigan State University, 612 Wilson Road, East Lansing, MI 48824*

‖*Renewable Resources and Enabling Sciences Center, National Renewable Energy Laboratory, 15013 Denver West Parkway, Golden, CO 80401*

E-mail: vermaasj@msu.edu; michael.crowley@nrel.gov; gregg.beckham@nrel.gov

Supplementary Animation 1 shows the full permeation and transit for a single syringol molecule as reported in Fig. 2 from the main text. Periodic images for the membrane are shown, with some trajectory smoothing applied to reduce the potential for motion-sickness in the reader. LPS polysaccharides are shown in yellow, while lipid components are shown

in gray. The syringol molecule uses the standard color scheme of white for hydrogen, gray for carbon, and red for oxygen atoms.

Table S1: Partition (P) and Permeability (Pm) coefficients for the LRCs enumerated in Fig. 1 in the OM mimic. The permeability coefficient of crossing the entire membrane, going from aqueous solution to aqueous solution, is decomposed into a crossing permeability (Pm_c) and extraction permeabilities into solution through the glycosylated (Pm_g) and unglycosylated (Pm_u) sides. The decomposition is done by adjusting the integrated bounds in Eq. 3 to integrate between free energy minima found near the lipid-water and lipid-glycosylation interfaces. This is described by Eq. 5 in the methods.

Group	Compound Name	$\log P$	$\log Pm\,(cms^{-1})$	$\log Pm_u\,(cms^{-1})$	$\log Pm_c\,(cms^{-1})$	$\log Pm_g\,(cms^{-1})$
Phenols	Phenol	3.0	0.3	-0.9	0.1	-2.7
	Guaiacol	4.0	-0.8	-1.9	-0.3	-4.8
	Syringol	3.2	-1.0	-1.2	-0.2	-4.2
	Catechol	3.3	0.3	-1.2	-0.3	-3.0
Other Aromatics	Benzene	2.8	0.0	-0.9	1.9	-2.7
	Toluene	4.1	-0.7	-2.1	1.7	-4.8
	Styrene	3.2	-0.3	-1.3	1.6	-3.5
	o-Cresol	3.3	-0.6	-1.3	0.7	-3.9
	m-Cresol	4.3	-0.4	-2.2	0.4	-4.8
	p-Cresol	5.2	-1.0	-3.0	0.0	-6.2
Benzyl alcohols	4-Hydroxy-benzyl alcohol	1.4	-0.5	0.4	-1.7	-1.6
	Vanillyl alcohol	1.0	-0.4	0.8	-0.9	-1.3
	Syringyl alcohol	1.3	-1.6	0.6	-1.0	-2.9
	3,4-Dihydroxy-benzyl alcohol	1.9	-0.6	0.2	-2.0	-2.4
Benzaldehydes	p-Hydroxy-benzaldehyde	1.5	-0.2	0.5	-0.3	-1.6
	Vanillin	2.5	-1.6	-0.5	-1.4	-4.1
	Syringaldehyde	0.2	-1.2	1.2	-1.0	-1.2
	Protocatechuic aldehyde	2.4	-0.7	-0.4	-1.3	-3.1
Benzoates	p-Hydroxy-benzoate	-2.3	-13.0	2.3	-10.7	2.7
	Vanillate	1.3	-10.7	-0.9	-12.0	1.0
	Syringate	1.0	-11.6	-1.6	-12.6	1.5
	Protocatechuate	-1.7	-13.1	2.2	-11.4	2.0
Benzoic acids	p-Hydroxy benzoic acid	3.6	-0.5	-1.3	-0.6	-4.1
	Vanillic acid	1.9	-0.4	-0.1	-0.3	-2.3
	Syringic acid	1.5	-0.9	0.3	-0.5	-2.4
	Protocatechuic acid	4.0	-0.7	-1.8	-1.0	-4.7
Monolignols	p-Coumaryl alcohol	3.9	-1.9	-1.6	-0.9	-5.7
	Coniferyl alcohol	1.9	-1.0	-0.0	-0.4	-2.9
	Sinapyl alcohol	3.9	-2.9	-1.8	-1.3	-6.8
	Caffeyl alcohol	3.0	-0.7	-0.9	-2.0	-3.6
Monolignaldehydes	p-Coumaraldehyde	2.3	-0.4	-0.3	-1.6	-2.7
	Coniferaldehyde	0.8	-2.7	0.9	-1.1	-3.5
	Sinapaldehyde	-0.1	-2.5	1.3	-1.9	-2.2
	Caffealdehyde	3.3	-0.5	-1.3	-2.8	-3.8
Cinnamates	p-Coumarate	0.4	-10.0	0.0	-10.4	0.4
	Ferulate	1.5	-10.7	-2.4	-12.2	1.0
	Sinapate	2.2	-8.8	-3.0	-11.0	0.3
	Caffeate	-2.6	-11.9	2.3	-9.3	-0.5
Cinnamic acids	p-Coumaric acid	3.7	-0.3	-1.6	-3.6	-3.6
	Ferulic acid	2.2	-0.9	-0.5	-2.5	-3.0
	Sinapic acid	1.0	-1.8	0.7	-1.4	-2.7
	Caffeic acid	2.3	-1.1	-0.3	-2.5	-3.4

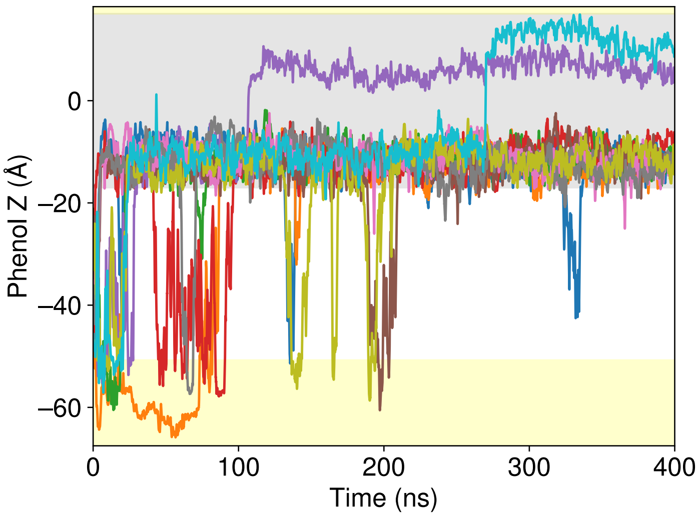

Figure S1: Trace plot analogous to Fig. 2 for phenol permeation. As in Fig. 2, each differently colored trace marks the pathway for a single molecule, which has been unwrapped across the periodic boundary to make it easier to follow visually. As an additional visual aid, regions corresponding to the outer membrane lipid core have a gray background, while glycosylated regions have a yellow background.

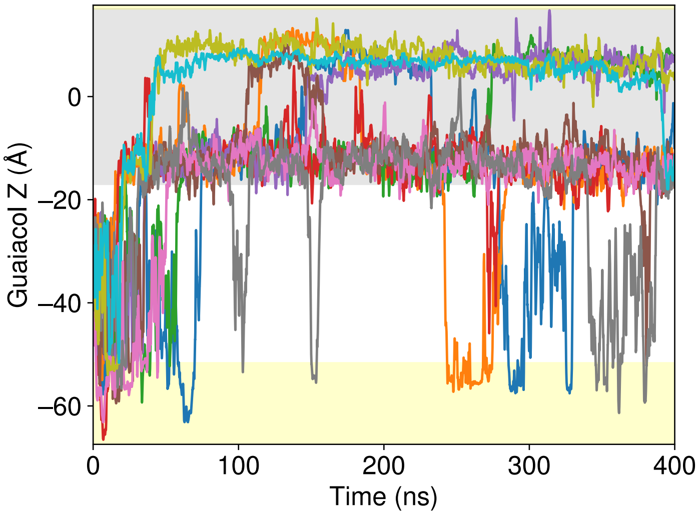

Figure S2: Trace plot analogous to Fig. 2 for guaiacol permeation. As in Fig. 2, each differently colored trace marks the pathway for a single molecule, which has been unwrapped across the periodic boundary to make it easier to follow visually. As an additional visual aid, regions corresponding to the outer membrane lipid core have a gray background, while glycosylated regions have a yellow background.

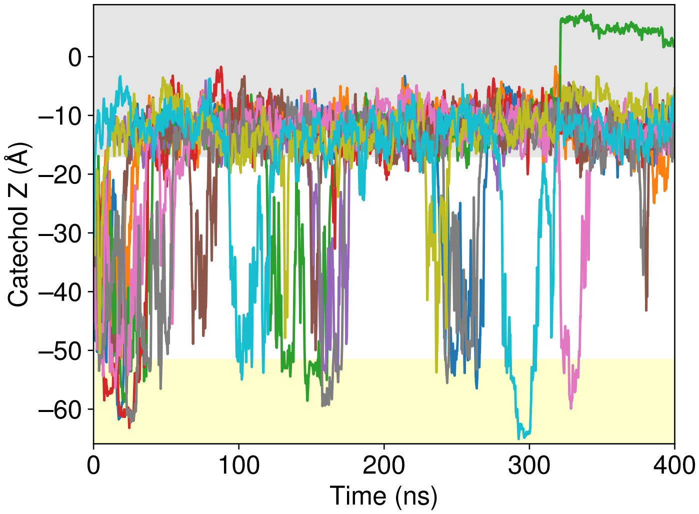

Figure S3: Trace plot analogous to Fig. 2 for catechol permeation. As in Fig. 2, each differently colored trace marks the pathway for a single molecule, which has been unwrapped across the periodic boundary to make it easier to follow visually. As an additional visual aid, regions corresponding to the outer membrane lipid core have a gray background, while glycosylated regions have a yellow background.

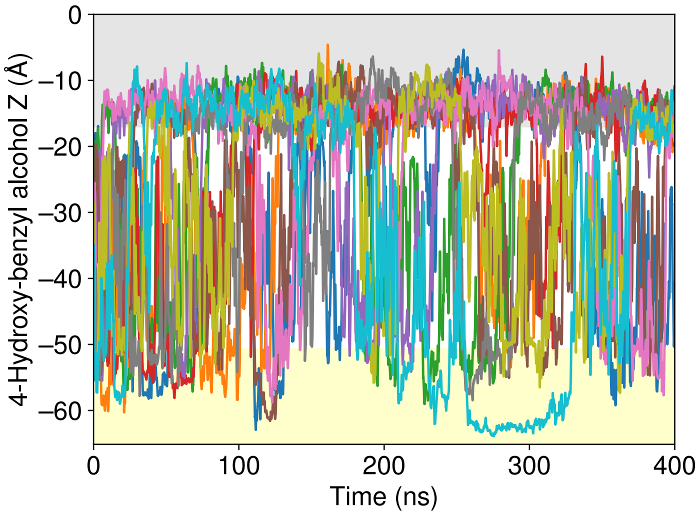

Figure S4: Trace plot analogous to Fig. 2 for 4-hydroxy-benzyl alcohol permeation. As in Fig. 2, each differently colored trace marks the pathway for a single molecule, which has been unwrapped across the periodic boundary to make it easier to follow visually. As an additional visual aid, regions corresponding to the outer membrane lipid core have a gray background, while glycosylated regions have a yellow background.

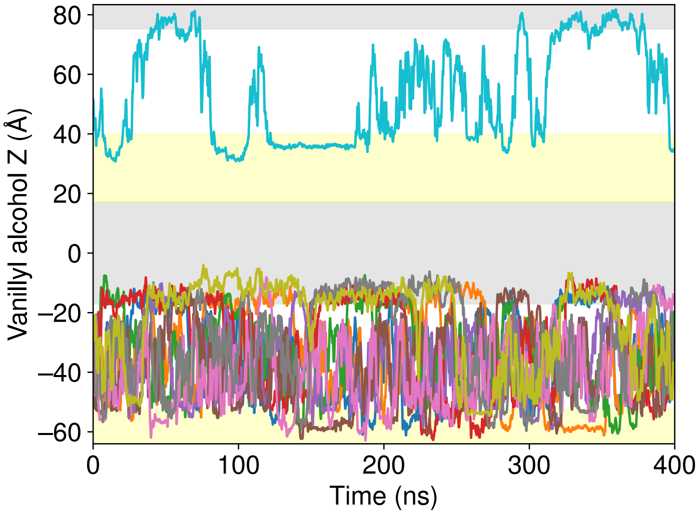

Figure S5: Trace plot analogous to Fig. 2 for vanillyl alcohol permeation. As in Fig. 2, each differently colored trace marks the pathway for a single molecule, which has been unwrapped across the periodic boundary to make it easier to follow visually. As an additional visual aid, regions corresponding to the outer membrane lipid core have a gray background, while glycosylated regions have a yellow background.

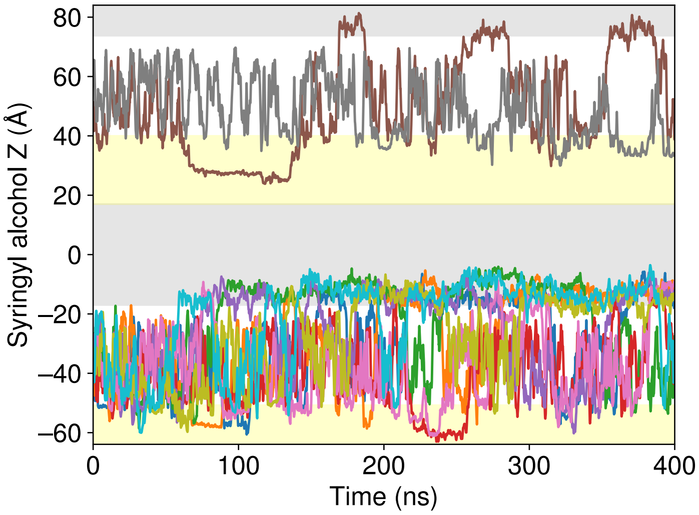

Figure S6: Trace plot analogous to Fig. 2 for syringyl alcohol permeation. As in Fig. 2, each differently colored trace marks the pathway for a single molecule, which has been unwrapped across the periodic boundary to make it easier to follow visually. As an additional visual aid, regions corresponding to the outer membrane lipid core have a gray background, while glycosylated regions have a yellow background.

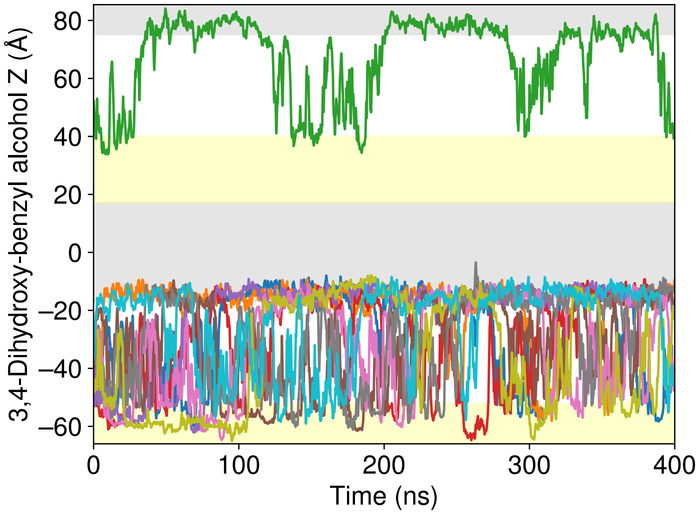

Figure S7: Trace plot analogous to Fig. 2 for 3,4-dihydroxy-benzyl alcohol permeation. As in Fig. 2, each differently colored trace marks the pathway for a single molecule, which has been unwrapped across the periodic boundary to make it easier to follow visually. As an additional visual aid, regions corresponding to the outer membrane lipid core have a gray background, while glycosylated regions have a yellow background.

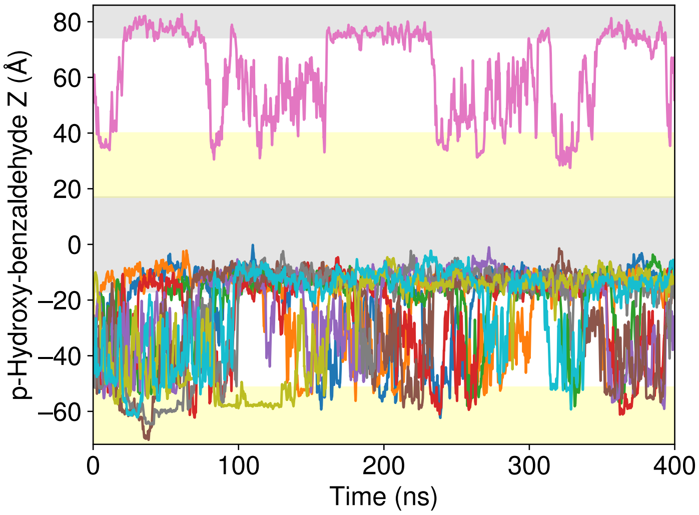

Figure S8: Trace plot analogous to Fig. 2 for p-hydroxy-benzaldehyde permeation. As in Fig. 2, each differently colored trace marks the pathway for a single molecule, which has been unwrapped across the periodic boundary to make it easier to follow visually. As an additional visual aid, regions corresponding to the outer membrane lipid core have a gray background, while glycosylated regions have a yellow background.

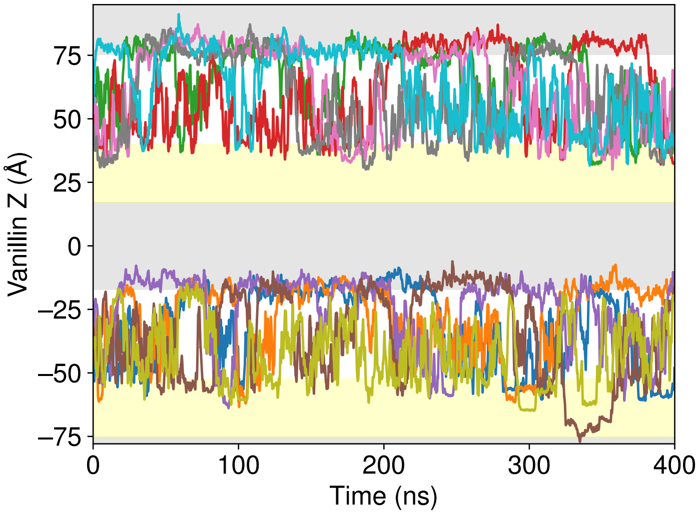

Figure S9: Trace plot analogous to Fig. 2 for vanillin permeation. As in Fig. 2, each differently colored trace marks the pathway for a single molecule, which has been unwrapped across the periodic boundary to make it easier to follow visually. As an additional visual aid, regions corresponding to the outer membrane lipid core have a gray background, while glycosylated regions have a yellow background.

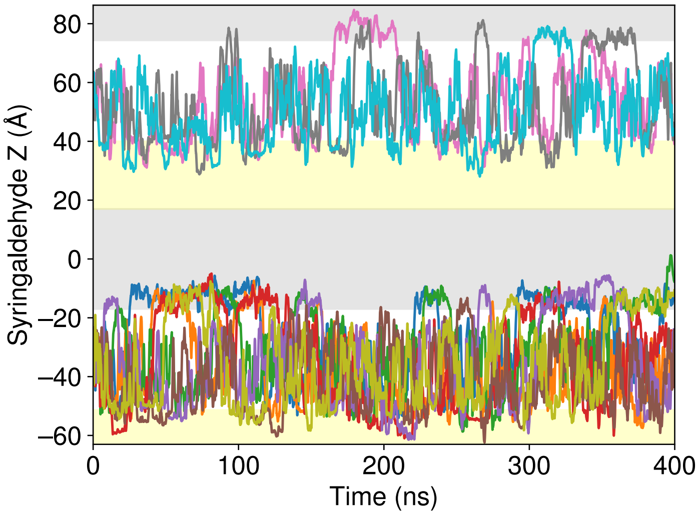

Figure S10: Trace plot analogous to Fig. 2 for syringaldehyde permeation. As in Fig. 2, each differently colored trace marks the pathway for a single molecule, which has been unwrapped across the periodic boundary to make it easier to follow visually. As an additional visual aid, regions corresponding to the outer membrane lipid core have a gray background, while glycosylated regions have a yellow background.

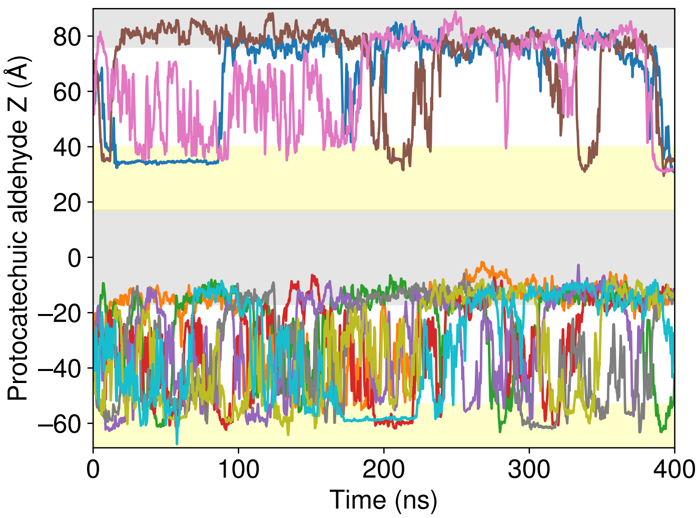

Figure S11: Trace plot analogous to Fig. 2 for protocatechuic aldehyde permeation. As in Fig. 2, each differently colored trace marks the pathway for a single molecule, which has been unwrapped across the periodic boundary to make it easier to follow visually. As an additional visual aid, regions corresponding to the outer membrane lipid core have a gray background, while glycosylated regions have a yellow background.

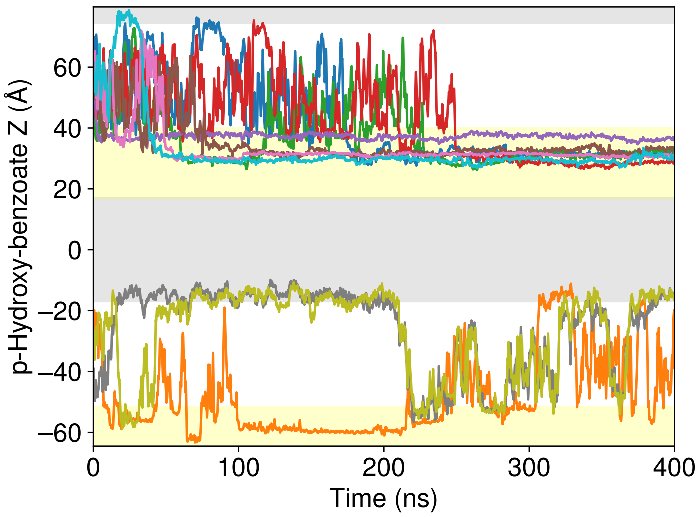

Figure S12: Trace plot analogous to Fig. 2 for p-hydroxy-benzoate permeation. As in Fig. 2, each differently colored trace marks the pathway for a single molecule, which has been unwrapped across the periodic boundary to make it easier to follow visually. As an additional visual aid, regions corresponding to the outer membrane lipid core have a gray background, while glycosylated regions have a yellow background.

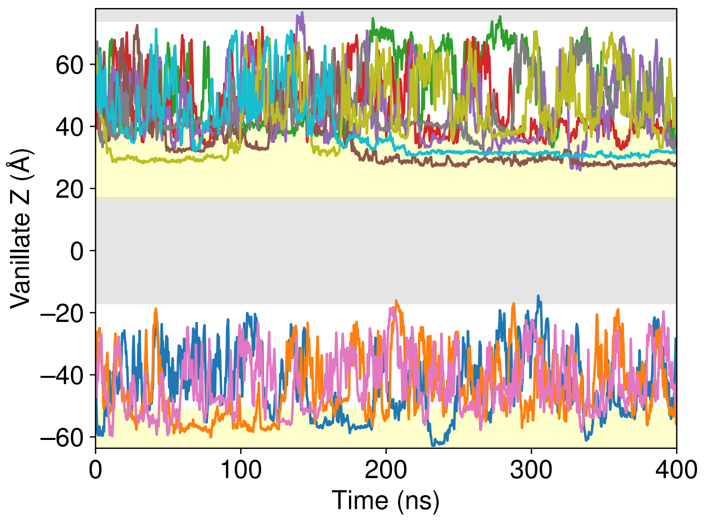

Figure S13: Trace plot analogous to Fig. 2 for vanillate permeation. As in Fig. 2, each differently colored trace marks the pathway for a single molecule, which has been unwrapped across the periodic boundary to make it easier to follow visually. As an additional visual aid, regions corresponding to the outer membrane lipid core have a gray background, while glycosylated regions have a yellow background.

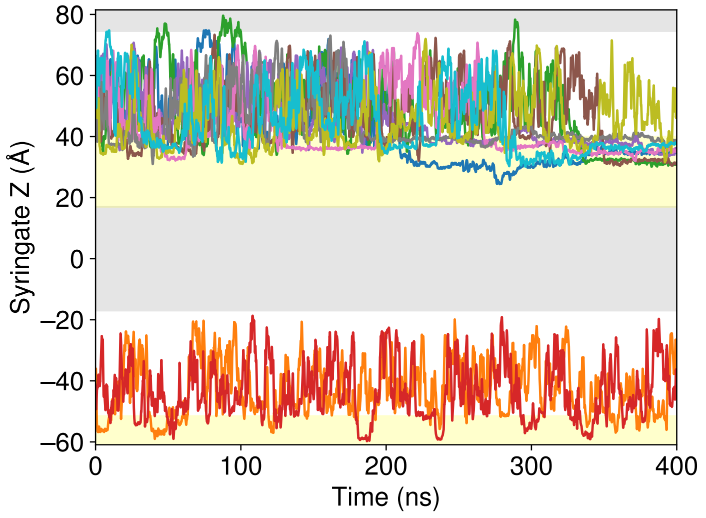

Figure S14: Trace plot analogous to Fig. 2 for syringate permeation. As in Fig. 2, each differently colored trace marks the pathway for a single molecule, which has been unwrapped across the periodic boundary to make it easier to follow visually. As an additional visual aid, regions corresponding to the outer membrane lipid core have a gray background, while glycosylated regions have a yellow background.

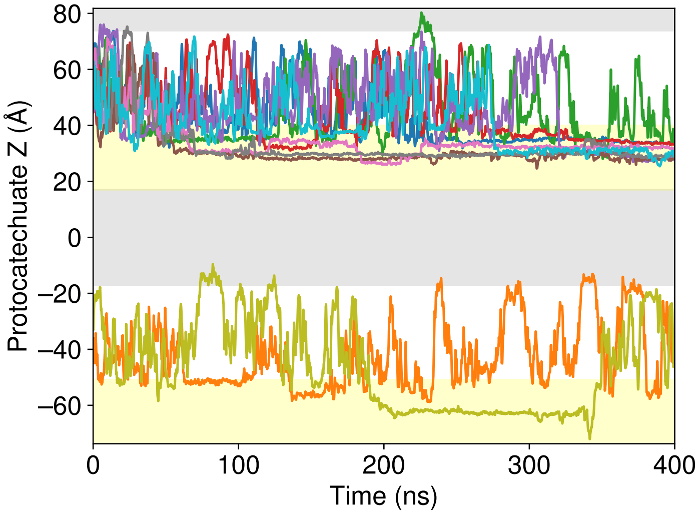

Figure S15: Trace plot analogous to Fig. 2 for protocatechuate permeation. As in Fig. 2, each differently colored trace marks the pathway for a single molecule, which has been unwrapped across the periodic boundary to make it easier to follow visually. As an additional visual aid, regions corresponding to the outer membrane lipid core have a gray background, while glycosylated regions have a yellow background.

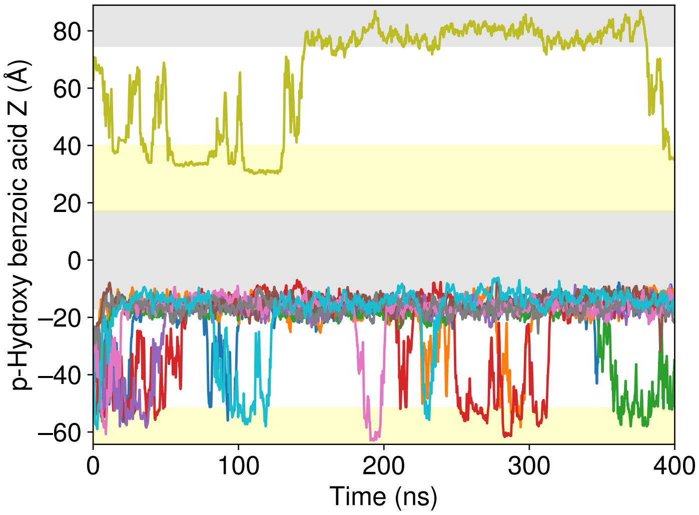

Figure S16: Trace plot analogous to Fig. 2 for p-hydroxy benzoic acid permeation. As in Fig. 2, each differently colored trace marks the pathway for a single molecule, which has been unwrapped across the periodic boundary to make it easier to follow visually. As an additional visual aid, regions corresponding to the outer membrane lipid core have a gray background, while glycosylated regions have a yellow background.

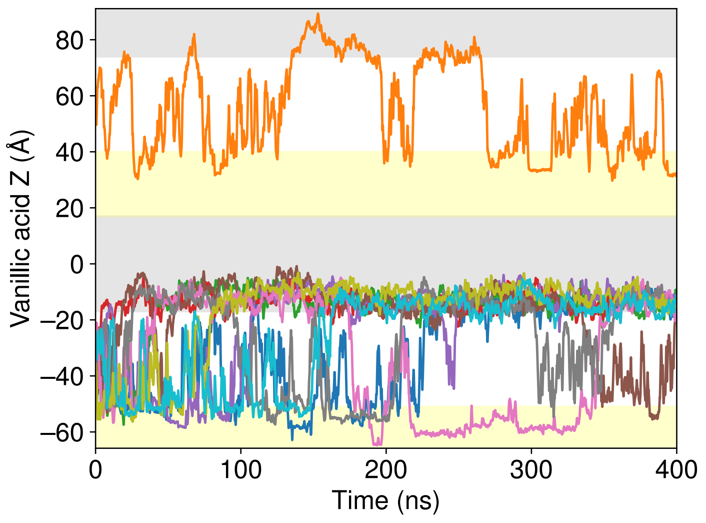

Figure S17: Trace plot analogous to Fig. 2 for vanillic acid permeation. As in Fig. 2, each differently colored trace marks the pathway for a single molecule, which has been unwrapped across the periodic boundary to make it easier to follow visually. As an additional visual aid, regions corresponding to the outer membrane lipid core have a gray background, while glycosylated regions have a yellow background.

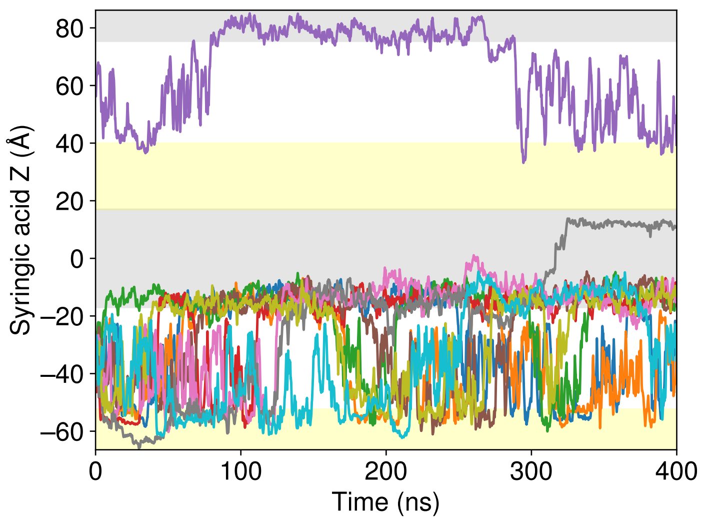

Figure S18: Trace plot analogous to Fig. 2 for syringic acid permeation. As in Fig. 2, each differently colored trace marks the pathway for a single molecule, which has been unwrapped across the periodic boundary to make it easier to follow visually. As an additional visual aid, regions corresponding to the outer membrane lipid core have a gray background, while glycosylated regions have a yellow background.

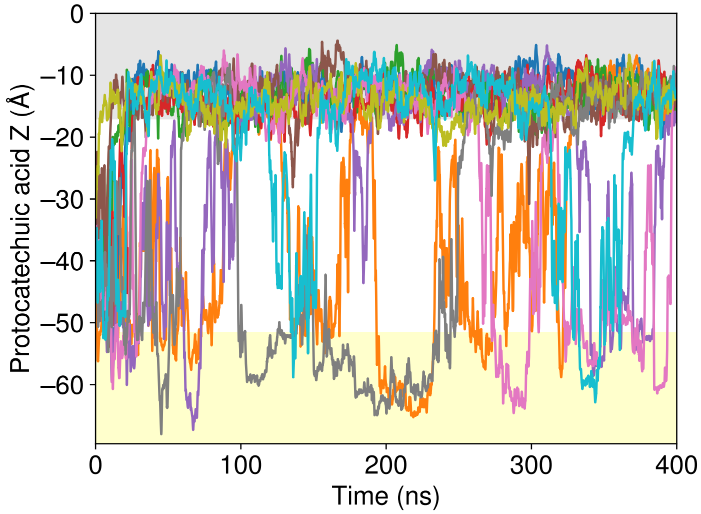

Figure S19: Trace plot analogous to Fig. 2 for protocatechuic acid permeation. As in Fig. 2, each differently colored trace marks the pathway for a single molecule, which has been unwrapped across the periodic boundary to make it easier to follow visually. As an additional visual aid, regions corresponding to the outer membrane lipid core have a gray background, while glycosylated regions have a yellow background.

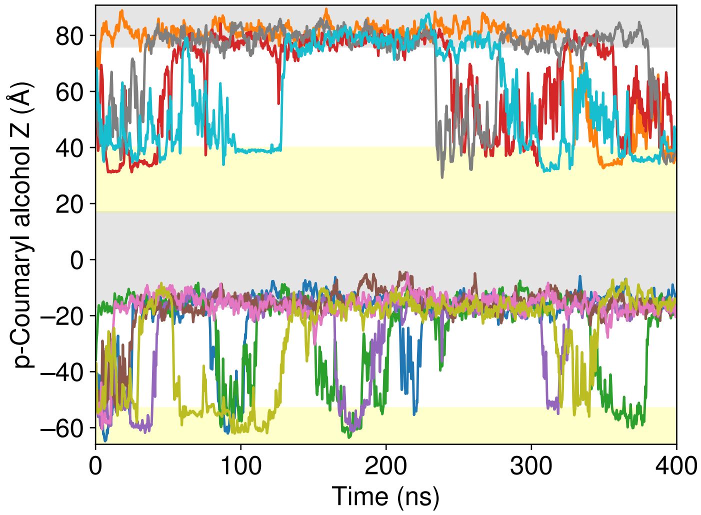

Figure S20: Trace plot analogous to Fig. 2 for p-coumaryl alcohol permeation. As in Fig. 2, each differently colored trace marks the pathway for a single molecule, which has been unwrapped across the periodic boundary to make it easier to follow visually. As an additional visual aid, regions corresponding to the outer membrane lipid core have a gray background, while glycosylated regions have a yellow background.

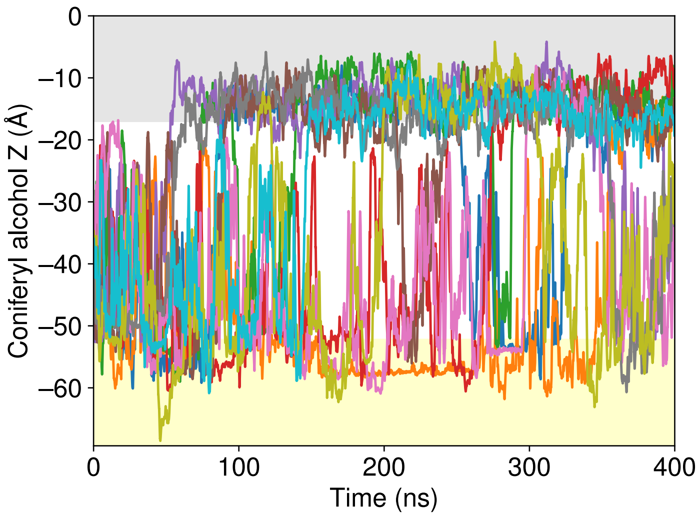

Figure S21: Trace plot analogous to Fig. 2 for coniferyl alcohol permeation. As in Fig. 2, each differently colored trace marks the pathway for a single molecule, which has been unwrapped across the periodic boundary to make it easier to follow visually. As an additional visual aid, regions corresponding to the outer membrane lipid core have a gray background, while glycosylated regions have a yellow background.

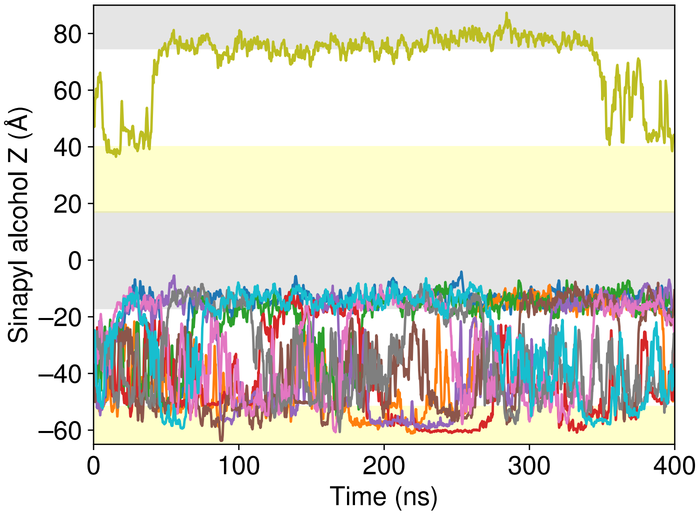

Figure S22: Trace plot analogous to Fig. 2 for sinapyl alcohol permeation. As in Fig. 2, each differently colored trace marks the pathway for a single molecule, which has been unwrapped across the periodic boundary to make it easier to follow visually. As an additional visual aid, regions corresponding to the outer membrane lipid core have a gray background, while glycosylated regions have a yellow background.

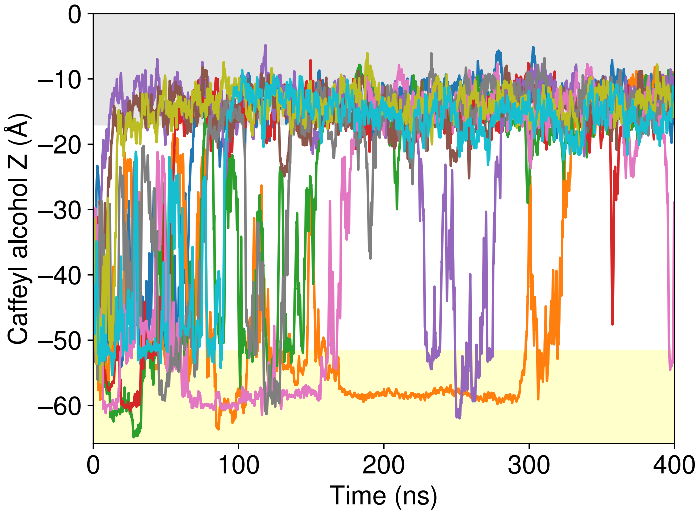

Figure S23: Trace plot analogous to Fig. 2 for caffeyl alcohol permeation. As in Fig. 2, each differently colored trace marks the pathway for a single molecule, which has been unwrapped across the periodic boundary to make it easier to follow visually. As an additional visual aid, regions corresponding to the outer membrane lipid core have a gray background, while glycosylated regions have a yellow background.

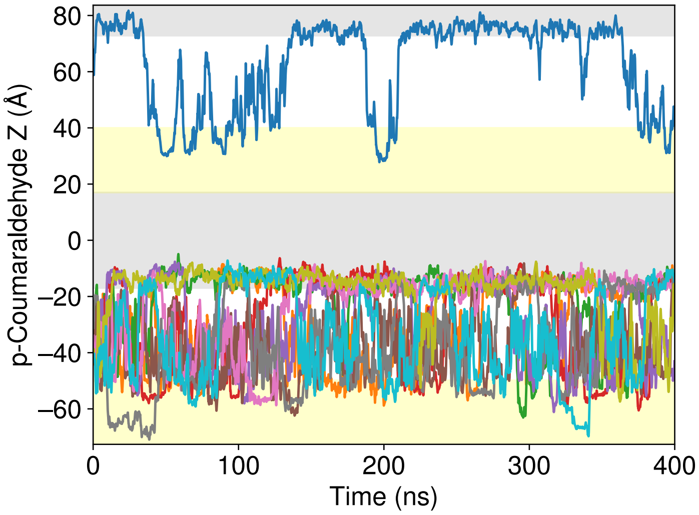

Figure S24: Trace plot analogous to Fig. 2 for p-coumaraldehyde permeation. As in Fig. 2, each differently colored trace marks the pathway for a single molecule, which has been unwrapped across the periodic boundary to make it easier to follow visually. As an additional visual aid, regions corresponding to the outer membrane lipid core have a gray background, while glycosylated regions have a yellow background.

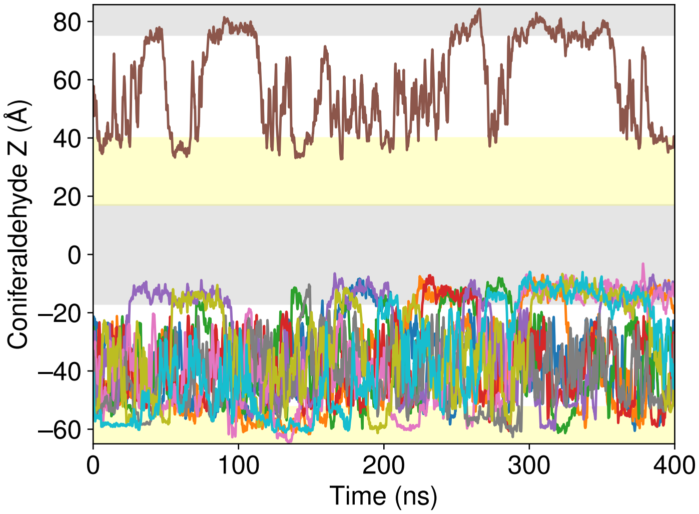

Figure S25: Trace plot analogous to Fig. 2 for coniferaldehyde permeation. As in Fig. 2, each differently colored trace marks the pathway for a single molecule, which has been unwrapped across the periodic boundary to make it easier to follow visually. As an additional visual aid, regions corresponding to the outer membrane lipid core have a gray background, while glycosylated regions have a yellow background.

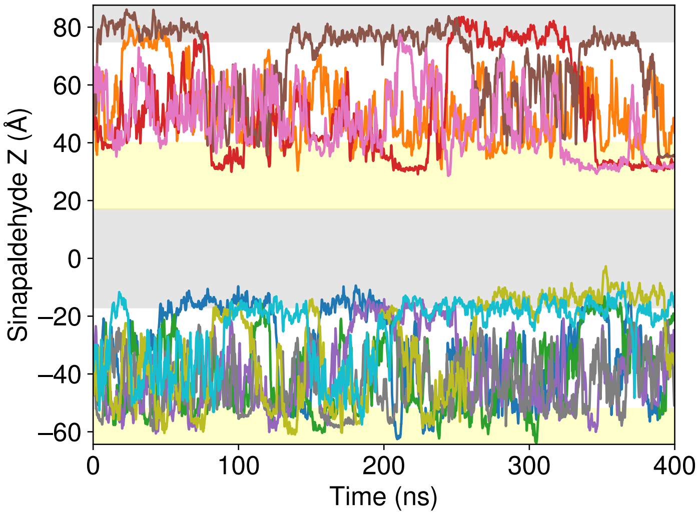

Figure S26: Trace plot analogous to Fig. 2 for sinapaldehyde permeation. As in Fig. 2, each differently colored trace marks the pathway for a single molecule, which has been unwrapped across the periodic boundary to make it easier to follow visually. As an additional visual aid, regions corresponding to the outer membrane lipid core have a gray background, while glycosylated regions have a yellow background.

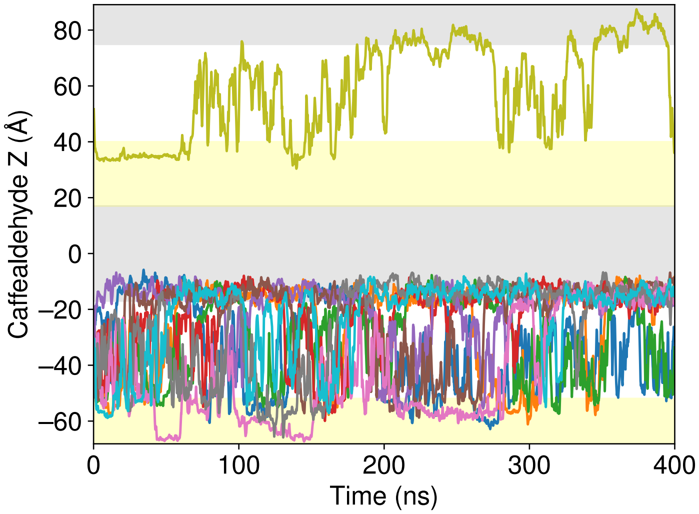

Figure S27: Trace plot analogous to Fig. 2 for caffealdehyde permeation. As in Fig. 2, each differently colored trace marks the pathway for a single molecule, which has been unwrapped across the periodic boundary to make it easier to follow visually. As an additional visual aid, regions corresponding to the outer membrane lipid core have a gray background, while glycosylated regions have a yellow background.

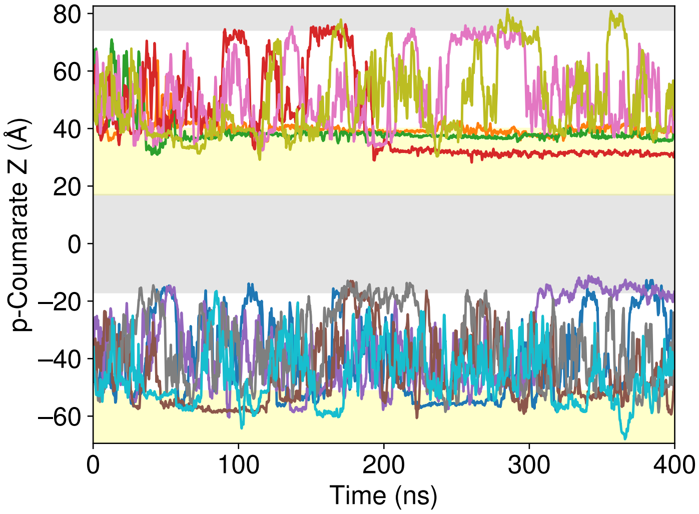

Figure S28: Trace plot analogous to Fig. 2 for p-coumarate permeation. As in Fig. 2, each differently colored trace marks the pathway for a single molecule, which has been unwrapped across the periodic boundary to make it easier to follow visually. As an additional visual aid, regions corresponding to the outer membrane lipid core have a gray background, while glycosylated regions have a yellow background.

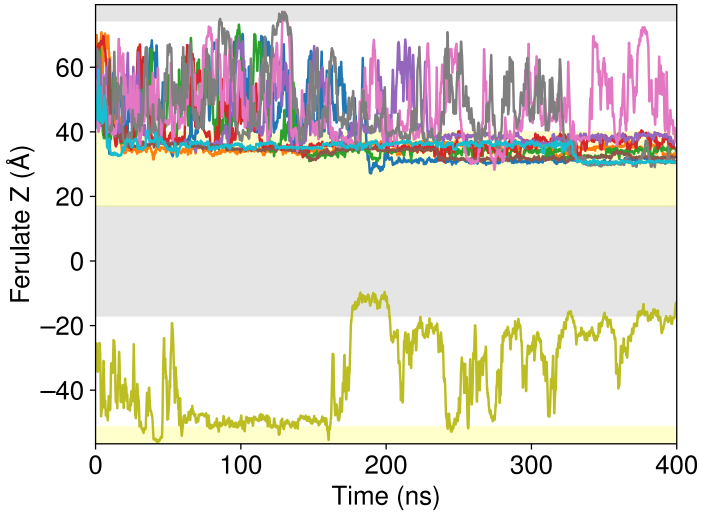

Figure S29: Trace plot analogous to Fig. 2 for ferulate permeation. As in Fig. 2, each differently colored trace marks the pathway for a single molecule, which has been unwrapped across the periodic boundary to make it easier to follow visually. As an additional visual aid, regions corresponding to the outer membrane lipid core have a gray background, while glycosylated regions have a yellow background.

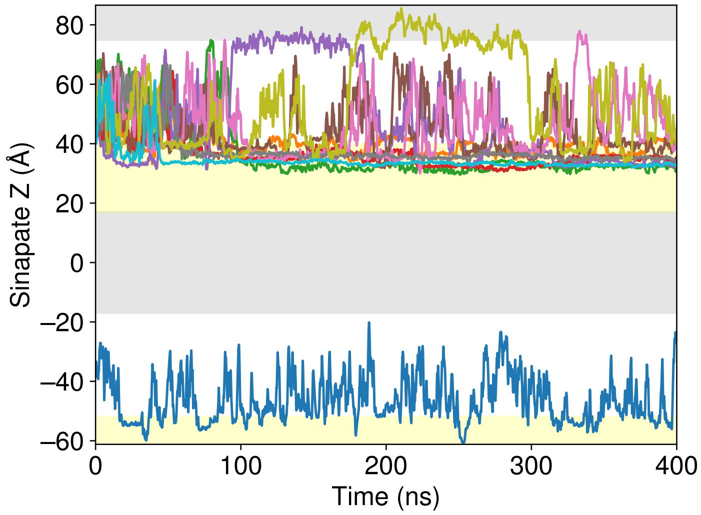

Figure S30: Trace plot analogous to Fig. 2 for sinapate permeation. As in Fig. 2, each differently colored trace marks the pathway for a single molecule, which has been unwrapped across the periodic boundary to make it easier to follow visually. As an additional visual aid, regions corresponding to the outer membrane lipid core have a gray background, while glycosylated regions have a yellow background.

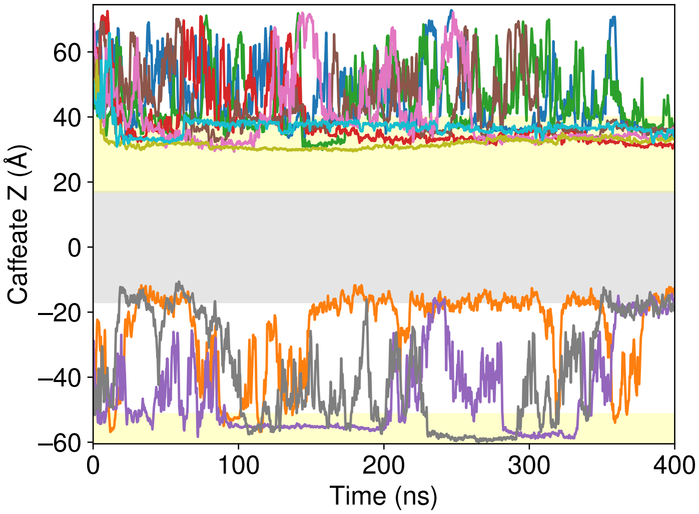

Figure S31: Trace plot analogous to Fig. 2 for caffeate permeation. As in Fig. 2, each differently colored trace marks the pathway for a single molecule, which has been unwrapped across the periodic boundary to make it easier to follow visually. As an additional visual aid, regions corresponding to the outer membrane lipid core have a gray background, while glycosylated regions have a yellow background.

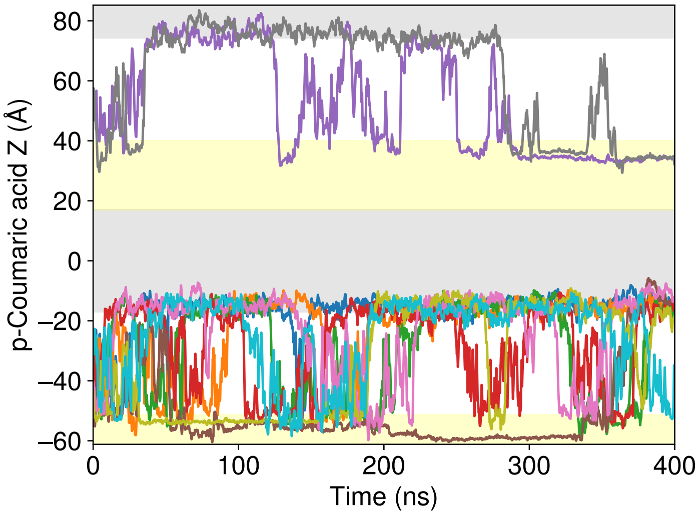

Figure S32: Trace plot analogous to Fig. 2 for p-coumaric acid permeation. As in Fig. 2, each differently colored trace marks the pathway for a single molecule, which has been unwrapped across the periodic boundary to make it easier to follow visually. As an additional visual aid, regions corresponding to the outer membrane lipid core have a gray background, while glycosylated regions have a yellow background.

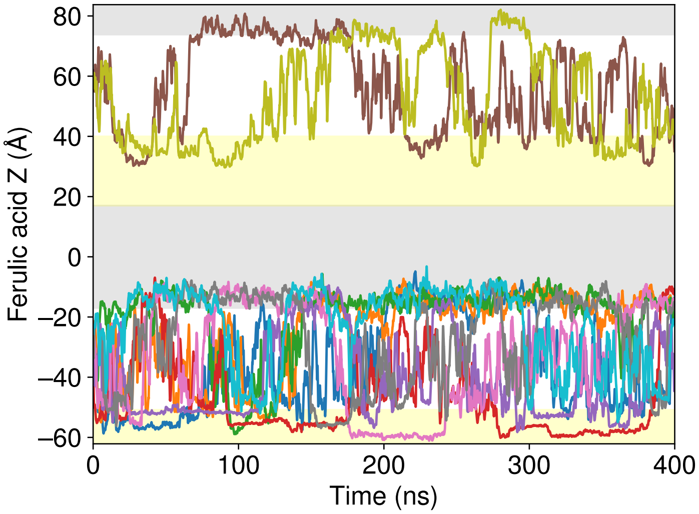

Figure S33: Trace plot analogous to Fig. 2 for ferulic acid permeation. As in Fig. 2, each differently colored trace marks the pathway for a single molecule, which has been unwrapped across the periodic boundary to make it easier to follow visually. As an additional visual aid, regions corresponding to the outer membrane lipid core have a gray background, while glycosylated regions have a yellow background.

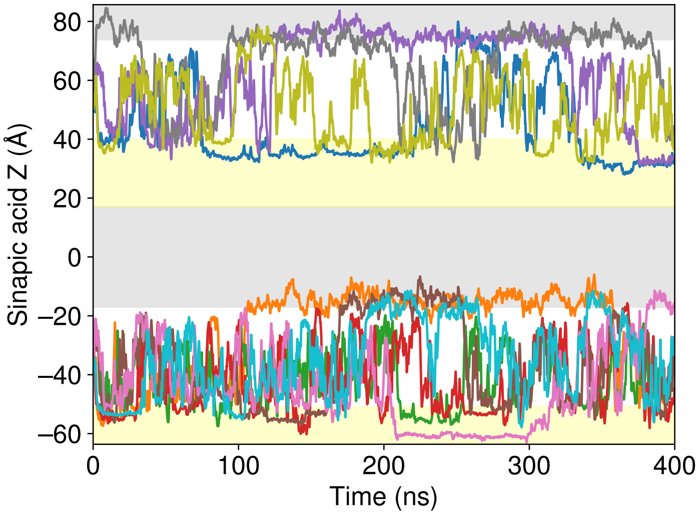

Figure S34: Trace plot analogous to Fig. 2 for sinapic acid permeation. As in Fig. 2, each differently colored trace marks the pathway for a single molecule, which has been unwrapped across the periodic boundary to make it easier to follow visually. As an additional visual aid, regions corresponding to the outer membrane lipid core have a gray background, while glycosylated regions have a yellow background.

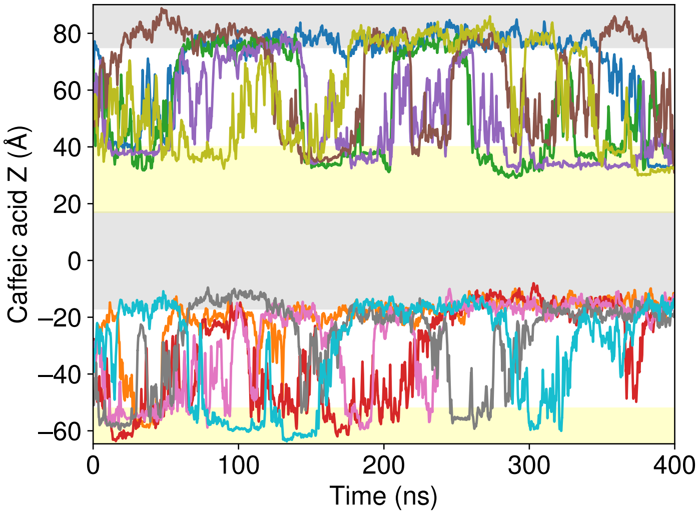

Figure S35: Trace plot analogous to Fig. 2 for caffeic acid permeation. As in Fig. 2, each differently colored trace marks the pathway for a single molecule, which has been unwrapped across the periodic boundary to make it easier to follow visually. As an additional visual aid, regions corresponding to the outer membrane lipid core have a gray background, while glycosylated regions have a yellow background.

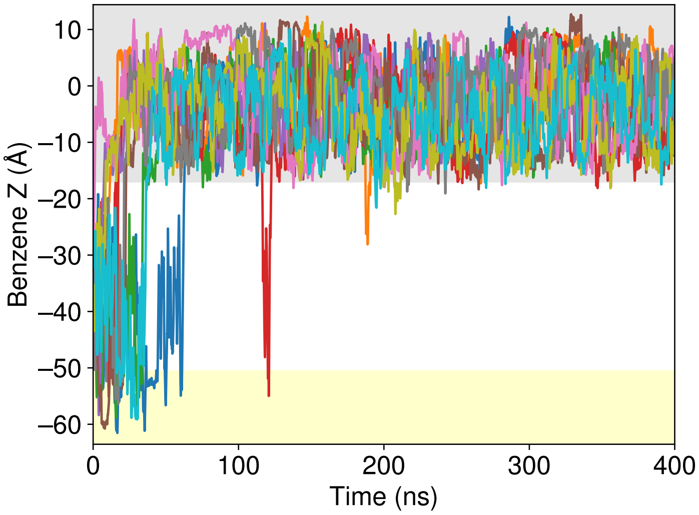

Figure S36: Trace plot analogous to Fig. 2 for benzene permeation. As in Fig. 2, each differently colored trace marks the pathway for a single molecule, which has been unwrapped across the periodic boundary to make it easier to follow visually. As an additional visual aid, regions corresponding to the outer membrane lipid core have a gray background, while glycosylated regions have a yellow background.

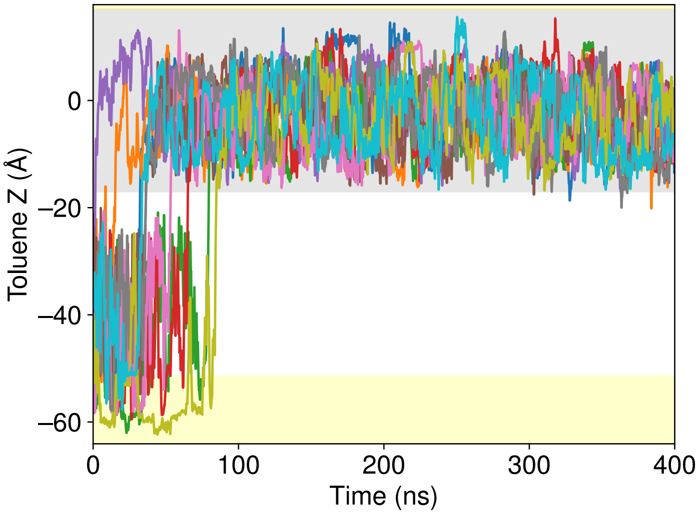

Figure S37: Trace plot analogous to Fig. 2 for toluene permeation. As in Fig. 2, each differently colored trace marks the pathway for a single molecule, which has been unwrapped across the periodic boundary to make it easier to follow visually. As an additional visual aid, regions corresponding to the outer membrane lipid core have a gray background, while glycosylated regions have a yellow background.

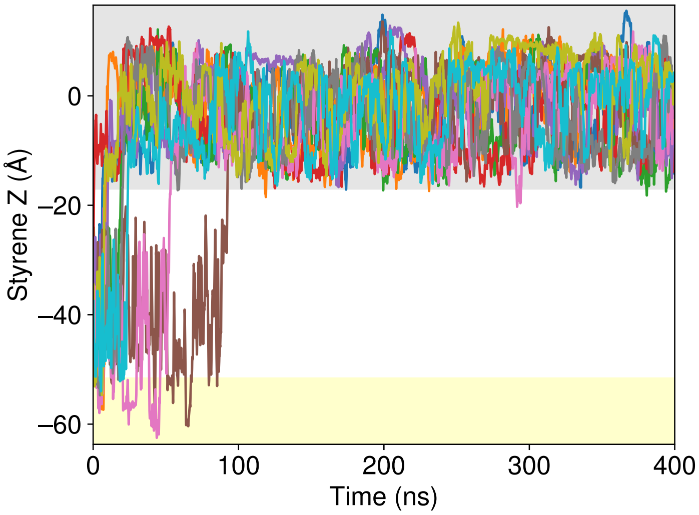

Figure S38: Trace plot analogous to Fig. 2 for styrene permeation. As in Fig. 2, each differently colored trace marks the pathway for a single molecule, which has been unwrapped across the periodic boundary to make it easier to follow visually. As an additional visual aid, regions corresponding to the outer membrane lipid core have a gray background, while glycosylated regions have a yellow background.

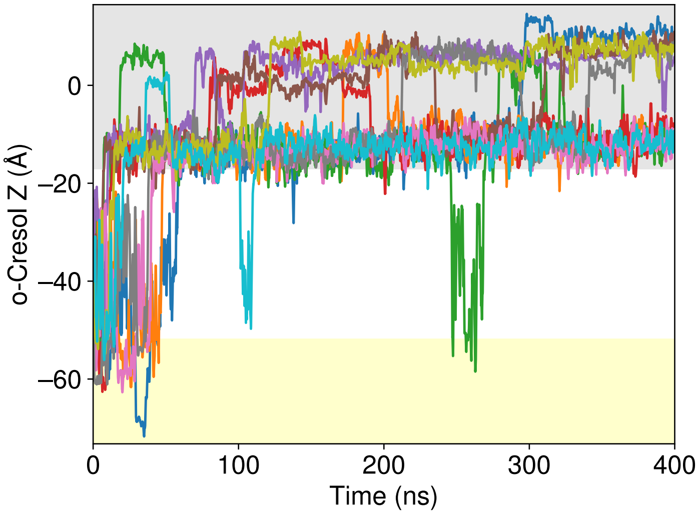

Figure S39: Trace plot analogous to Fig. 2 for o-cresol permeation. As in Fig. 2, each differently colored trace marks the pathway for a single molecule, which has been unwrapped across the periodic boundary to make it easier to follow visually. As an additional visual aid, regions corresponding to the outer membrane lipid core have a gray background, while glycosylated regions have a yellow background.

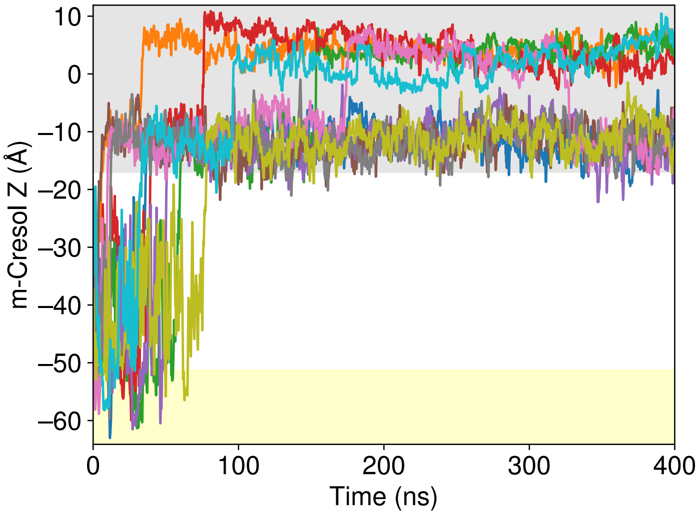

Figure S40: Trace plot analogous to Fig. 2 for m-cresol permeation. As in Fig. 2, each differently colored trace marks the pathway for a single molecule, which has been unwrapped across the periodic boundary to make it easier to follow visually. As an additional visual aid, regions corresponding to the outer membrane lipid core have a gray background, while glycosylated regions have a yellow background.

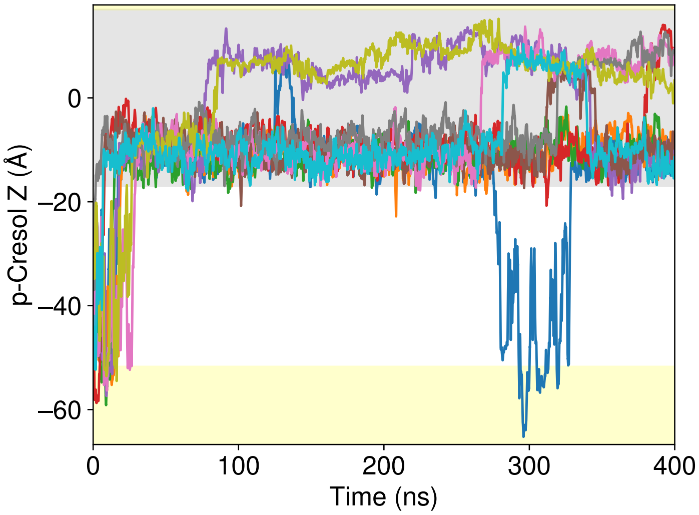

Figure S41: Trace plot analogous to Fig. 2 for p-cresol permeation. As in Fig. 2, each differently colored trace marks the pathway for a single molecule, which has been unwrapped across the periodic boundary to make it easier to follow visually. As an additional visual aid, regions corresponding to the outer membrane lipid core have a gray background, while glycosylated regions have a yellow background.

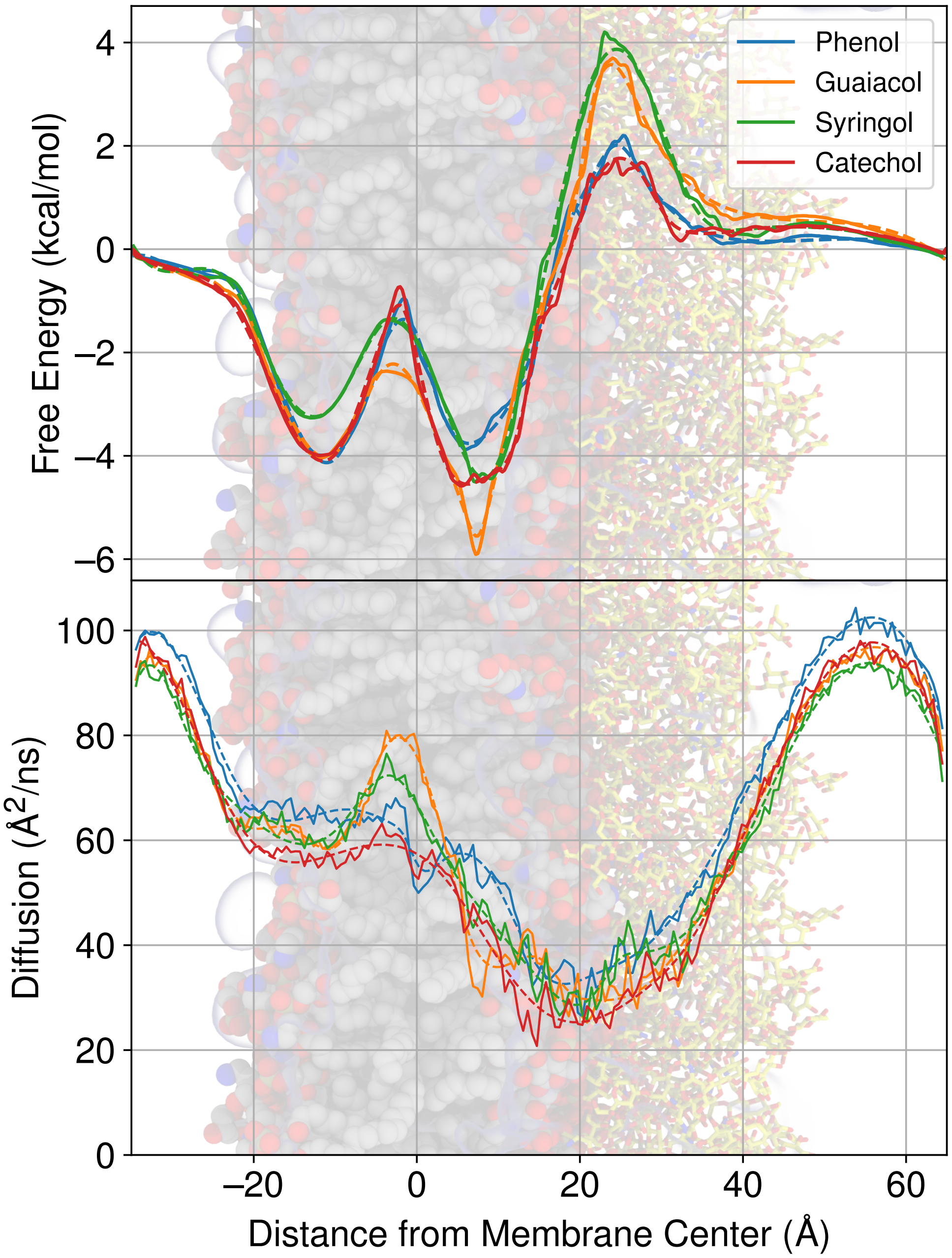

Figure S42: Free energy and diffusivity profiles analagous to Fig. 5 for phenols permeation. For context, the plots are underlaid with a molecular representation for the glycosylated membrane, following the color scheme from Fig. 10.

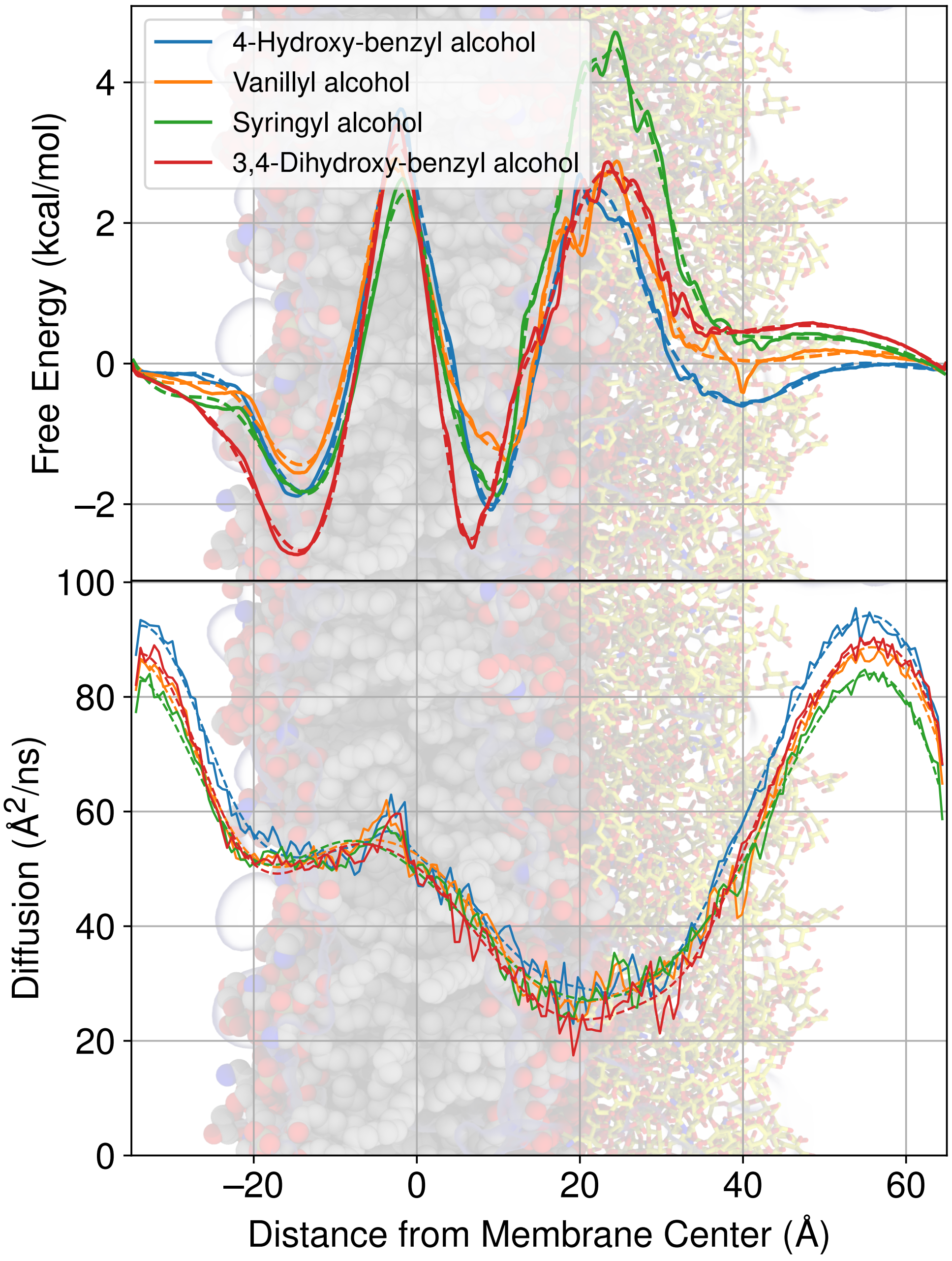

Figure S43: Free energy and diffusivity profiles analagous to Fig. 5 for benzyl alcohols permeation. For context, the plots are underlaid with a molecular representation for the glycosylated membrane, following the color scheme from Fig. 10.

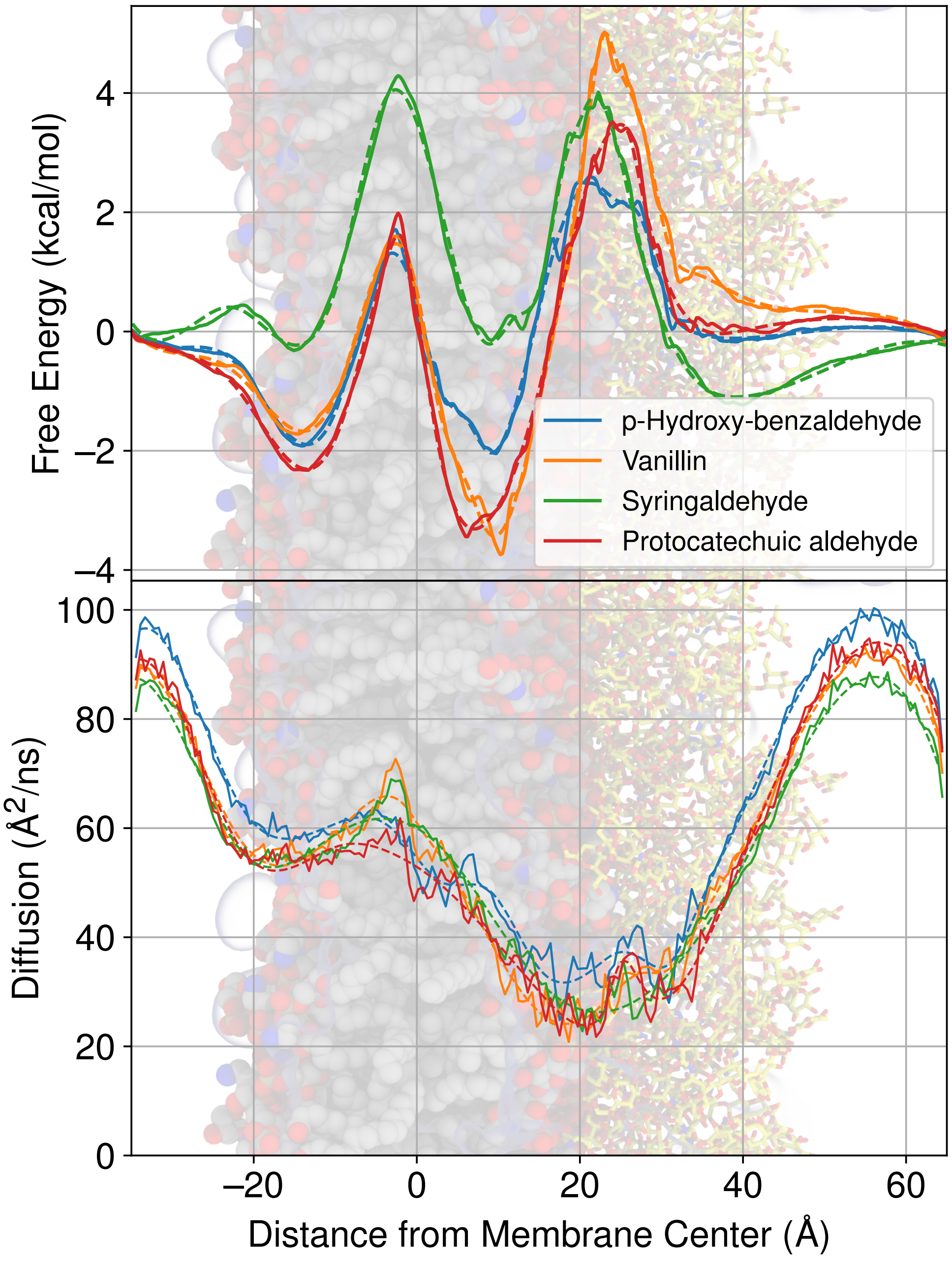

Figure S44: Free energy and diffusivity profiles analagous to Fig. 5 for benzaldehydes permeation. For context, the plots are underlaid with a molecular representation for the glycosylated membrane, following the color scheme from Fig. 10.

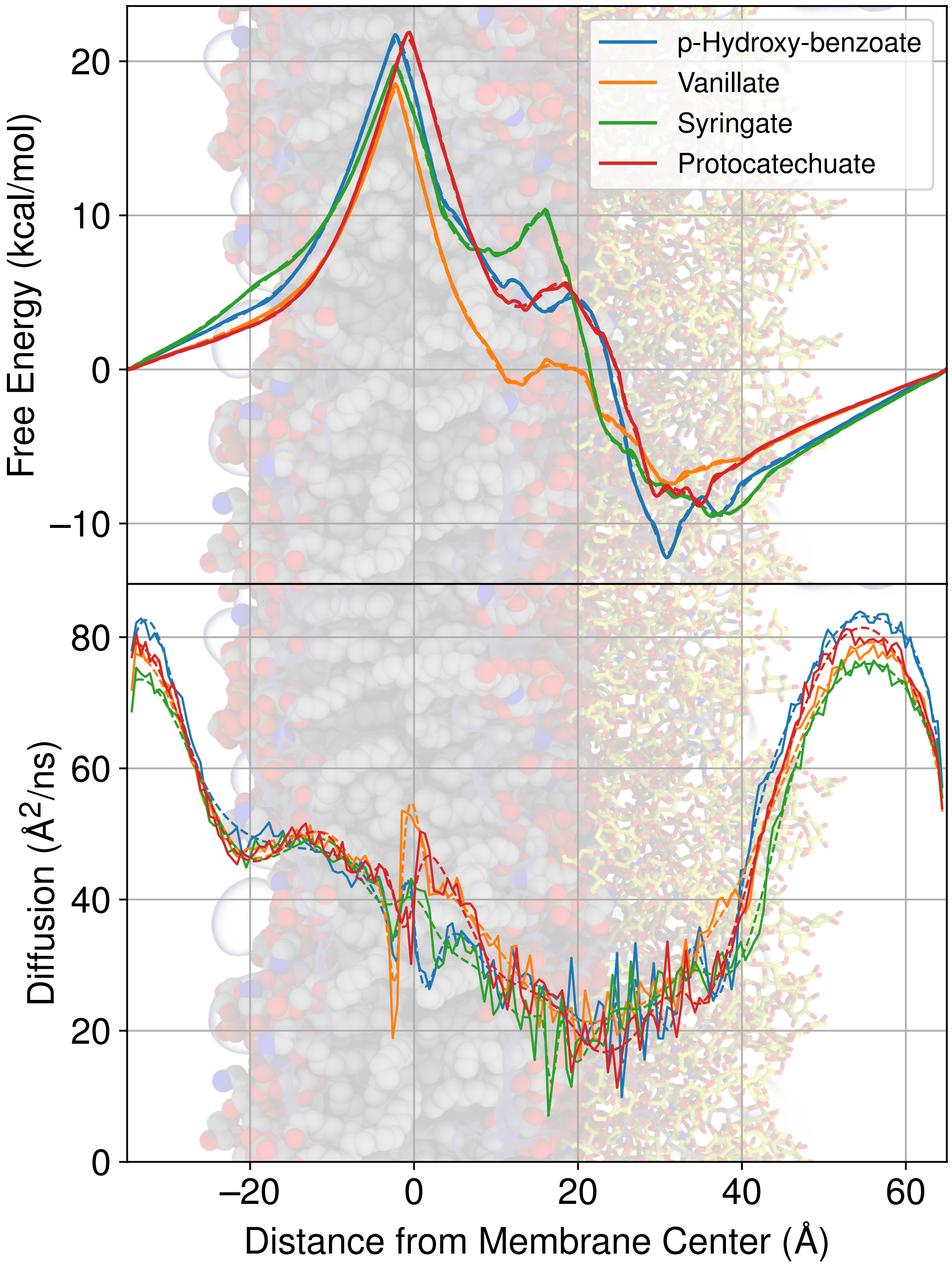

Figure S45: Free energy and diffusivity profiles analagous to Fig. 5 for benzoates permeation. For context, the plots are underlaid with a molecular representation for the glycosylated membrane, following the color scheme from Fig. 10.

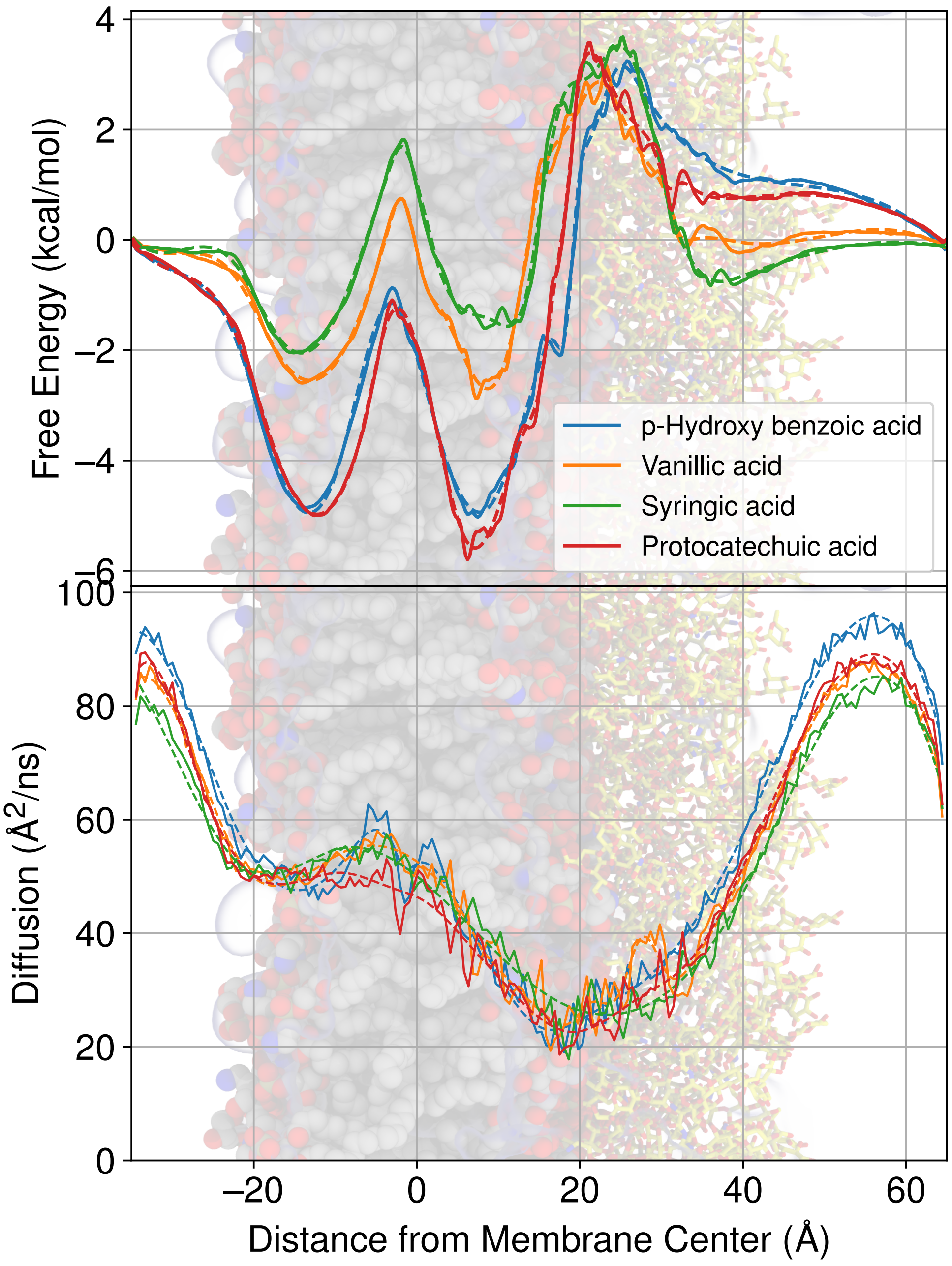

Figure S46: Free energy and diffusivity profiles analagous to Fig. 5 for benzoic acids permeation. For context, the plots are underlaid with a molecular representation for the glycosylated membrane, following the color scheme from Fig. 10.

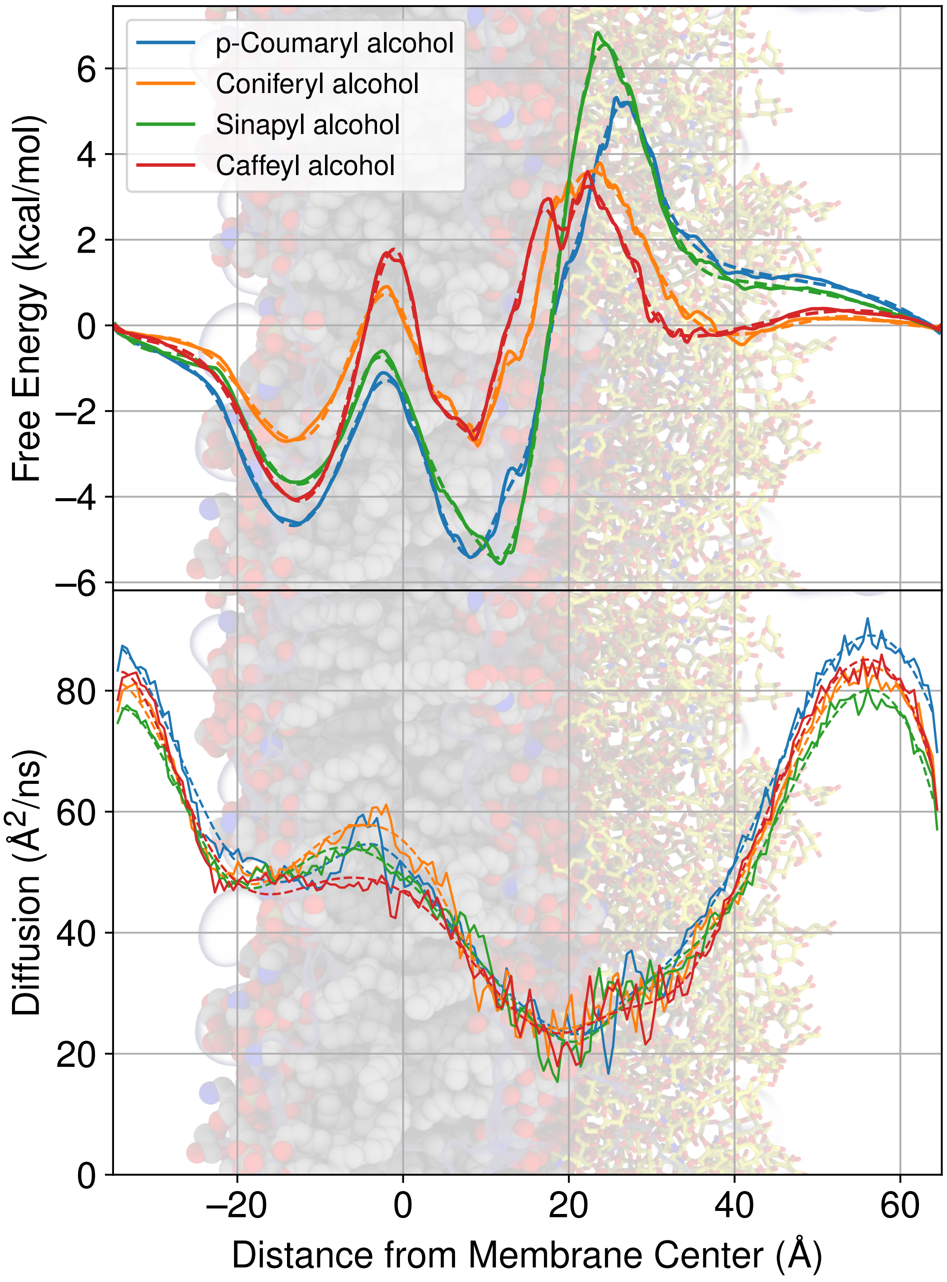

Figure S47: Free energy and diffusivity profiles analagous to Fig. 5 for monolignols permeation. For context, the plots are underlaid with a molecular representation for the glycosylated membrane, following the color scheme from Fig. 10.

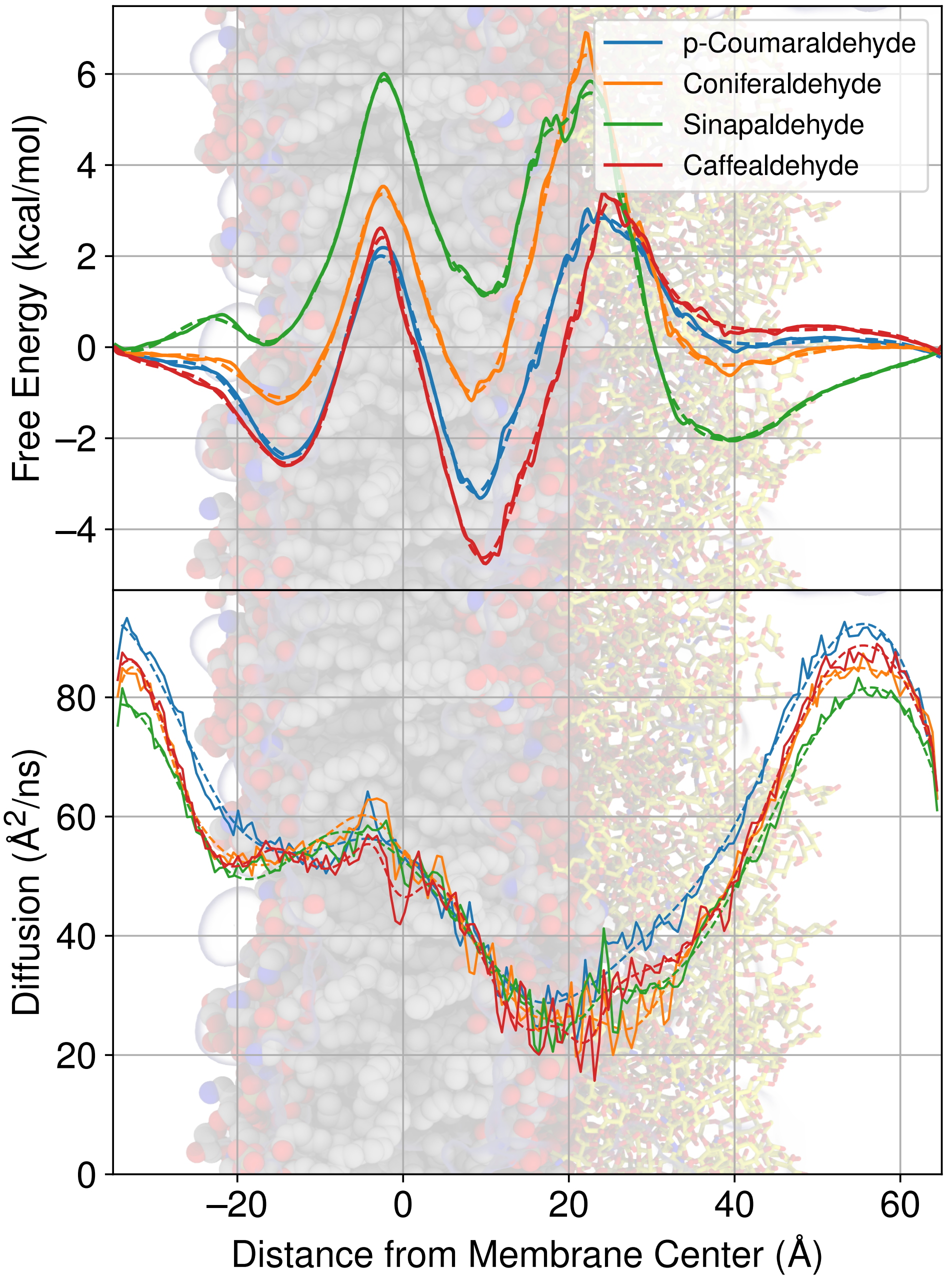

Figure S48: Free energy and diffusivity profiles analagous to Fig. 5 for monolignaldehydes permeation. For context, the plots are underlaid with a molecular representation for the glycosylated membrane, following the color scheme from Fig. 10.

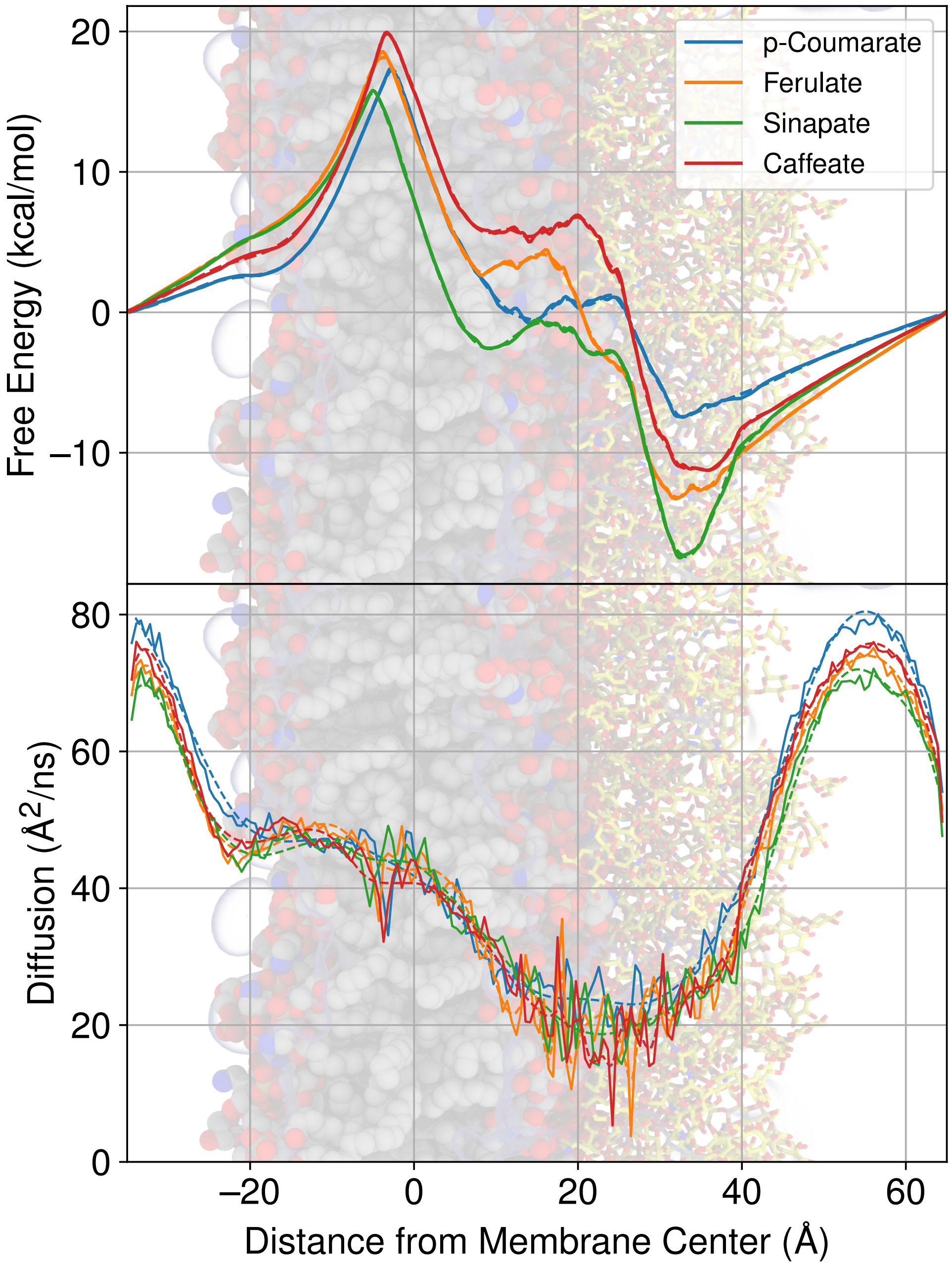

Figure S49: Free energy and diffusivity profiles analagous to Fig. 5 for cinnamates permeation. For context, the plots are underlaid with a molecular representation for the glycosylated membrane, following the color scheme from Fig. 10.

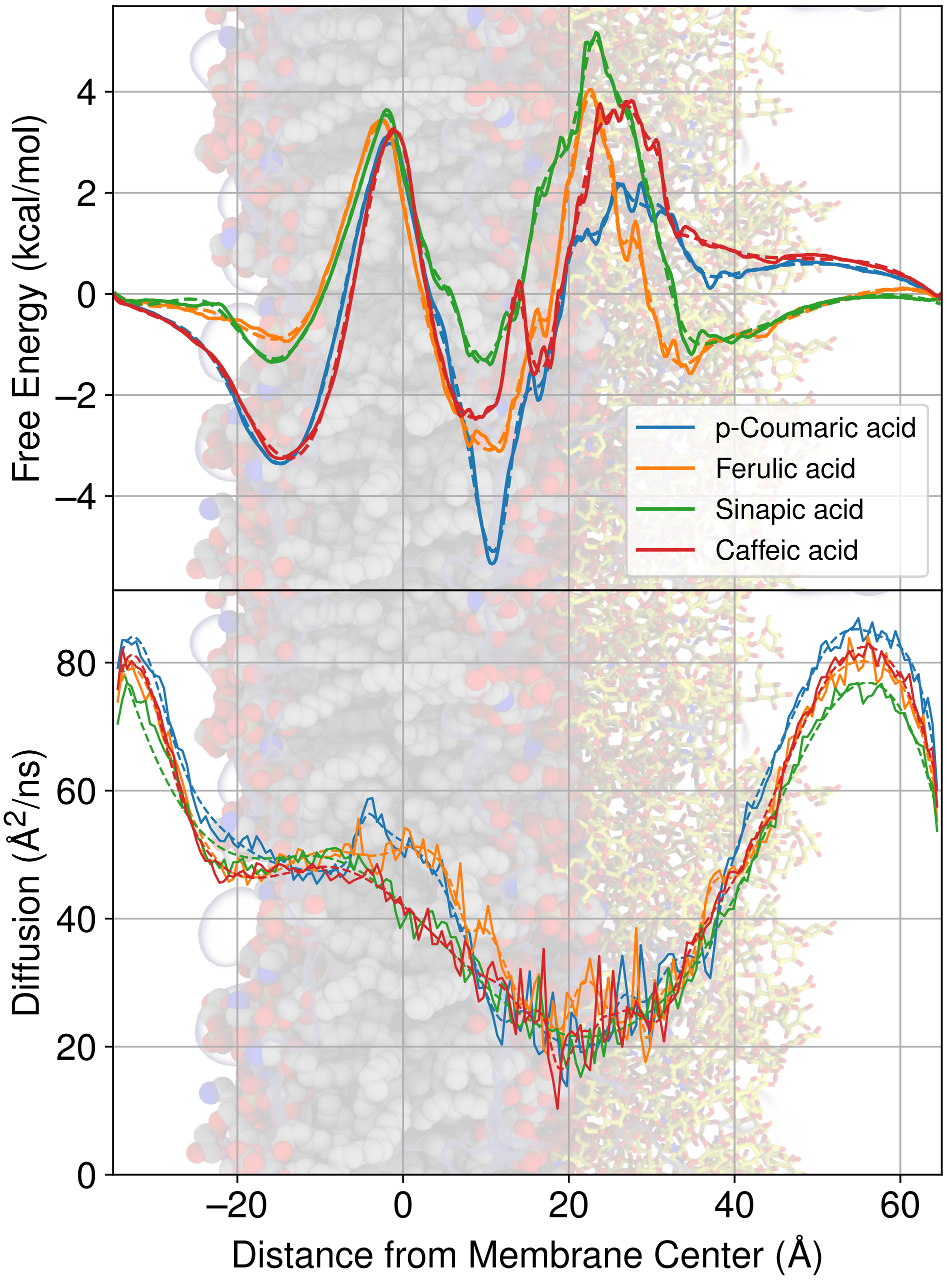

Figure S50: Free energy and diffusivity profiles analagous to Fig. 5 for cinnamic acids permeation. For context, the plots are underlaid with a molecular representation for the glycosylated membrane, following the color scheme from Fig. 10.

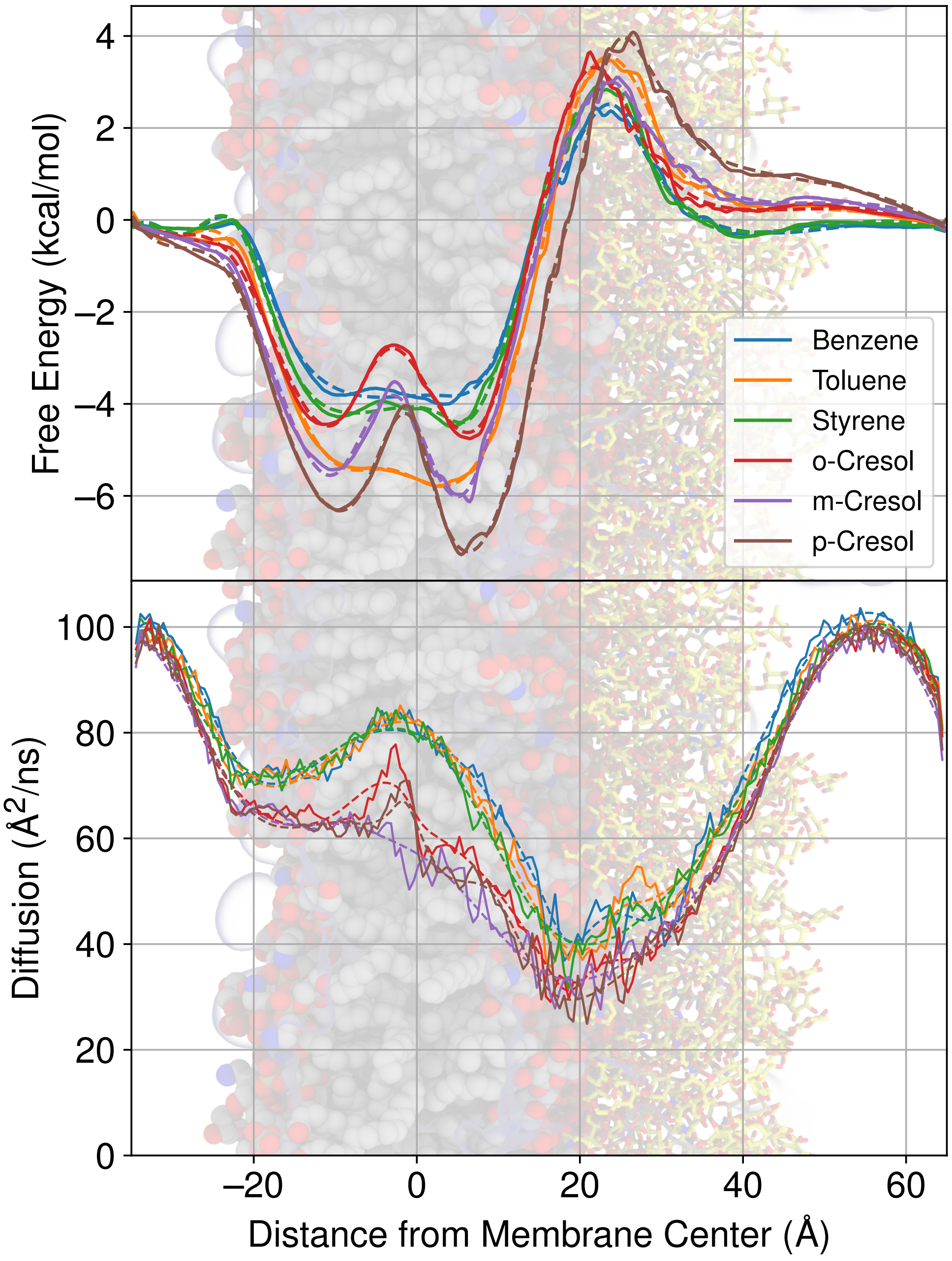

Figure S51: Free energy and diffusivity profiles analagous to Fig. 5 for other aromatics permeation. For context, the plots are underlaid with a molecular representation for the glycosylated membrane, following the color scheme from Fig. 10.

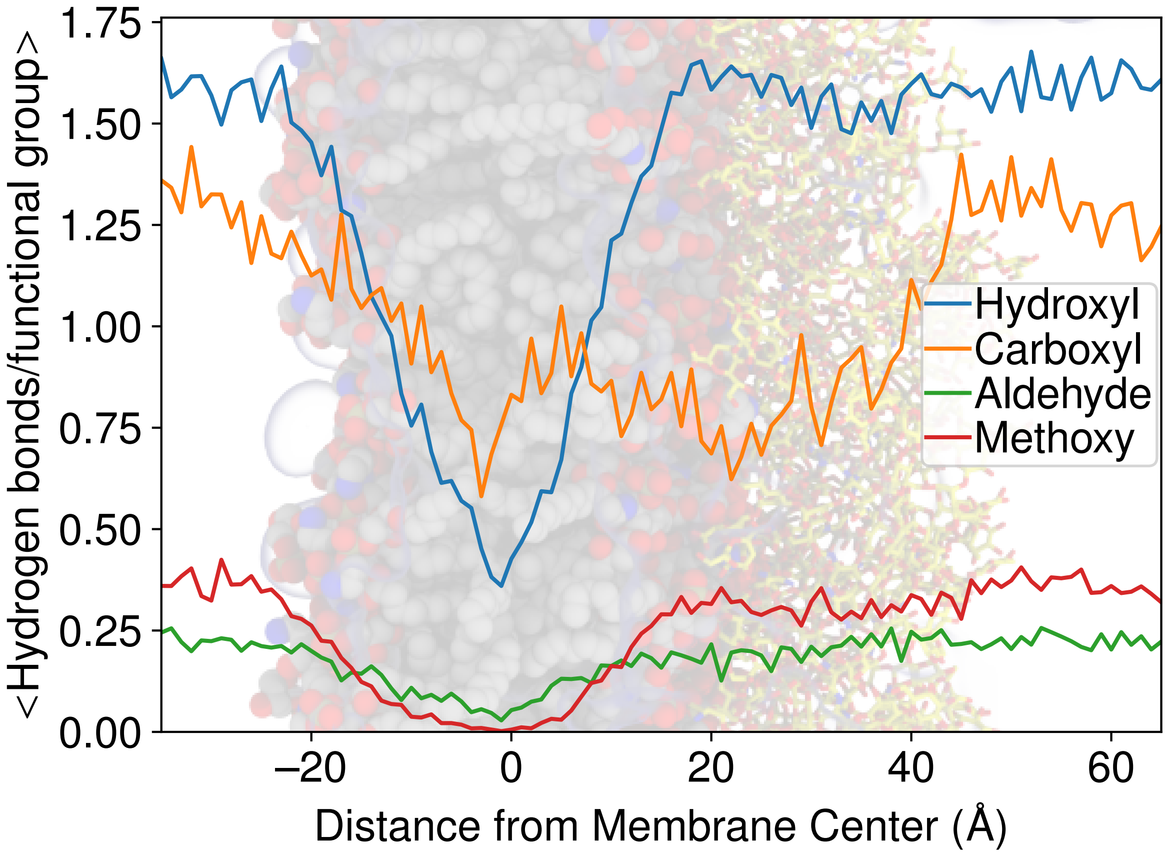

Figure S52: LRC-environment hydrogen bonds per LRC functional group within the REUS trajectories, plotted as a function of distance from the membrane center. This analysis considers the total number of hydrogen bonds per functional group within the LRC, defining a hydrogen bond by a donor-acceptor distance less than 3.2 Å and a hydrogen-donor-acceptor angles less than 30 degrees. The distance from the membrane center was computed from the center of mass for the LRC. To simplify computation, only frames corresponding to trajectory restarts were analyzed.
